# Targeting Ras-, Rho-, and Rab-family GTPases via a conserved cryptic pocket

**DOI:** 10.1016/j.cell.2024.08.017

**Published:** 2024-09-09

**Authors:** Johannes Morstein, Victoria Bowcut, Micah Fernando, Yue Yang, Lawrence Zhu, Meredith L. Jenkins, John T. Evans, Keelan Z. Guiley, D. Matthew Peacock, Sophie Krahnke, Zhi Lin, Katrine A. Taran, Benjamin J. Huang, Andrew G. Stephen, John E. Burke, Felice C. Lightstone, Kevan M. Shokat

**Affiliations:** 1 Department of Cellular and Molecular Pharmacology and Howard Hughes Medical Institute, University of California, San Francisco, CA 94158, USA; 2 Biosciences and Biotechnology Division, Physical and Life Sciences Directorate, Lawrence Livermore National Lab, Livermore, CA 94550, USA; 3 Department of Biochemistry and Microbiology, University of Victoria, Victoria, BC, Canada; 4 Department of Biochemistry and Molecular Biology, University of British Columbia, Vancouver, BC, Canada; 5 NCI RAS Initiative, Cancer Research Technology Program, Frederick National Laboratory for Cancer Research, Frederick, MD 21701, USA; 6 Department of Pharmaceutical Chemistry, University of California, San Francisco, CA 94158, USA; 7 Department of Pediatrics, University of California, San Francisco, CA 94158, USA; 8 Lead contact

## Abstract

The family of Ras-like GTPases consists of over 150 different members, regulated by an even larger number of guanine exchange factors (GEFs) and GTPase-activating proteins (GAPs) that comprise cellular switch networks that govern cell motility, growth, polarity, protein trafficking, and gene expression. Efforts to develop selective small molecule probes and drugs for these proteins have been hampered by the high affinity of guanosine triphosphate (GTP) and lack of allosteric regulatory sites. This paradigm was recently challenged by the discovery of a cryptic allosteric pocket in the switch II region of K-Ras. Here, we ask whether similar pockets are present in GTPases beyond K-Ras. We systematically surveyed members of the Ras, Rho, and Rab family of GTPases and found that many GTPases exhibit targetable switch II pockets. Notable differences in the composition and conservation of key residues offer potential for the development of optimized inhibitors for many members of this previously undruggable family.

## In brief

Morstein et al. demonstrate the feasibility of inhibiting GTPases beyond K-Ras, including various Ras-, Rho-, and Rab-family GTPases that were previously considered undruggable.

## Graphical abstract

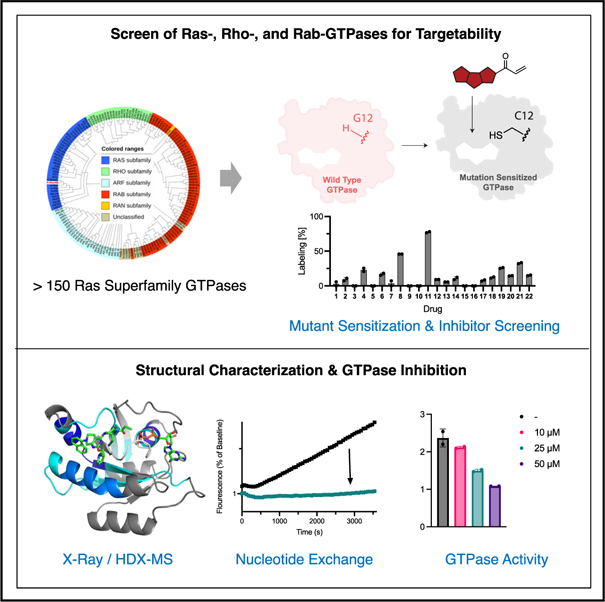

## INTRODUCTION

Together with their regulators and effectors, GTPases function as molecular switches that govern many fundamental cellular processes.^1,2^ The majority of these proteins belong to the Ras superfamily of small GTPases, which includes Ras, Rho, Rab, Arf, and Ran GTPases.^3–5^ Ras GTPases are involved in proliferation and migration, and their aberrant regulation is implicated in cancers and developmental diseases, termed RASopathies.^6,7^ Rho GTPases, including RhoA, Rac1, and Cdc42, control many aspects of actin dynamics underlying cytoskeletal organization and motility of cells.^8^ Rab GTPases are the largest subfamily and coordinate vesicular traffic.^9^ Arf GTPases are also critically involved in transport pathways, and Ran GTPases specifically coordinate nuclear transport.^10,11^ Small molecules that selectively target individual members of the GTPase superfamily could be valuable tools to dissect signaling function and enable the treatment of diseases that GTPases are implicated in. However, examples of such molecules are very limited and, unlike ATP-binding proteins, GTPases are still widely considered “undruggable” targets. This is due to the relatively tighter and phosphate-driven binding affinity of the nucleotide in GTPases compared with kinases and absence of allosteric sites in the apo proteins. Recently, the discovery of a cryptic allosteric pocket (switch II [SII] pocket) in K-Ras4B has challenged this paradigm and enabled the rapid development of therapeutics to target K-Ras(G12C) mutant cancers,^12,13^ including the FDA-approved drugs LUMAKRAS (sotorasib)^14^ and Krazati (adagrasib)^15^ for the treatment of non-small cell lung cancer. Other candidates, including GDC6036 (divarasib), are currently undergoing clinical trials and showing durable clinical responses.^16^ However, so far, K-Ras remains a unique case, and it is unclear whether other GTPases could be targeted in a similar fashion.

Herein, we systematically map SII pockets across members of various GTPase families and find that, despite limited sequence homology, key elements of the SII pocket are conserved across the Ras, Rho, and Rab families of GTPases, enabling the targeting of many GTPases beyond K-Ras. To study the ability of K-Ras(G12C) inhibitors to target other GTPases, we introduce the equivalent cysteine mutations to GTPases of interest. To the best of our knowledge, RabL5 is the only small GTPase (in the Ras superfamily) bearing a native cysteine in this position. As such, this approach has the potential to enable the selective inhibition of GTPases within a complex proteome in a manner similar to the chemical genetics approaches used to target protein kinases.^17,18^ Such a chemical genetics approach to GTPases would complement existing genetic methods and offer improved temporal resolution to offset for compensatory effects within complex networks like the Rab GTPase protein trafficking network, consisting of over 70 members. Although most existing chemical genetics approaches rely on co-factor pocket modulations, which are typically highly conserved, we are attempting to map and target allosteric sites across a large superfamily. This could not only present an additional challenge but also provide opportunities for selectivity and make the pocket more tractable for the future development of reversible inhibitors for various GTPases.

## RESULTS AND DISCUSSION

### K-Ras(G12C) inhibitors effectively target H-Ras(G12C) and N-Ras(G12C)

Since the discovery of an allosteric cryptic pocket in the SII region of K-Ras, several SII pocket inhibitors have been optimized to target K-Ras(G12C)-driven tumors.^13,19^ The SII pocket is not present in apo structures and occurs in the presence of a suitable ligand upon an outward movement of the relatively flexible SII region. In addition to the SII loop, several key residues engaging SII pocket ligands are found on the α3 helix, including H95, Y96, and Q99. These less-mobile residues likely make a considerable contribution to shaping the SII pocket. However, they are poorly conserved among small GTPases, which may present a significant challenge in targeting other GTPases beyond K-Ras.

**Figure 1.**
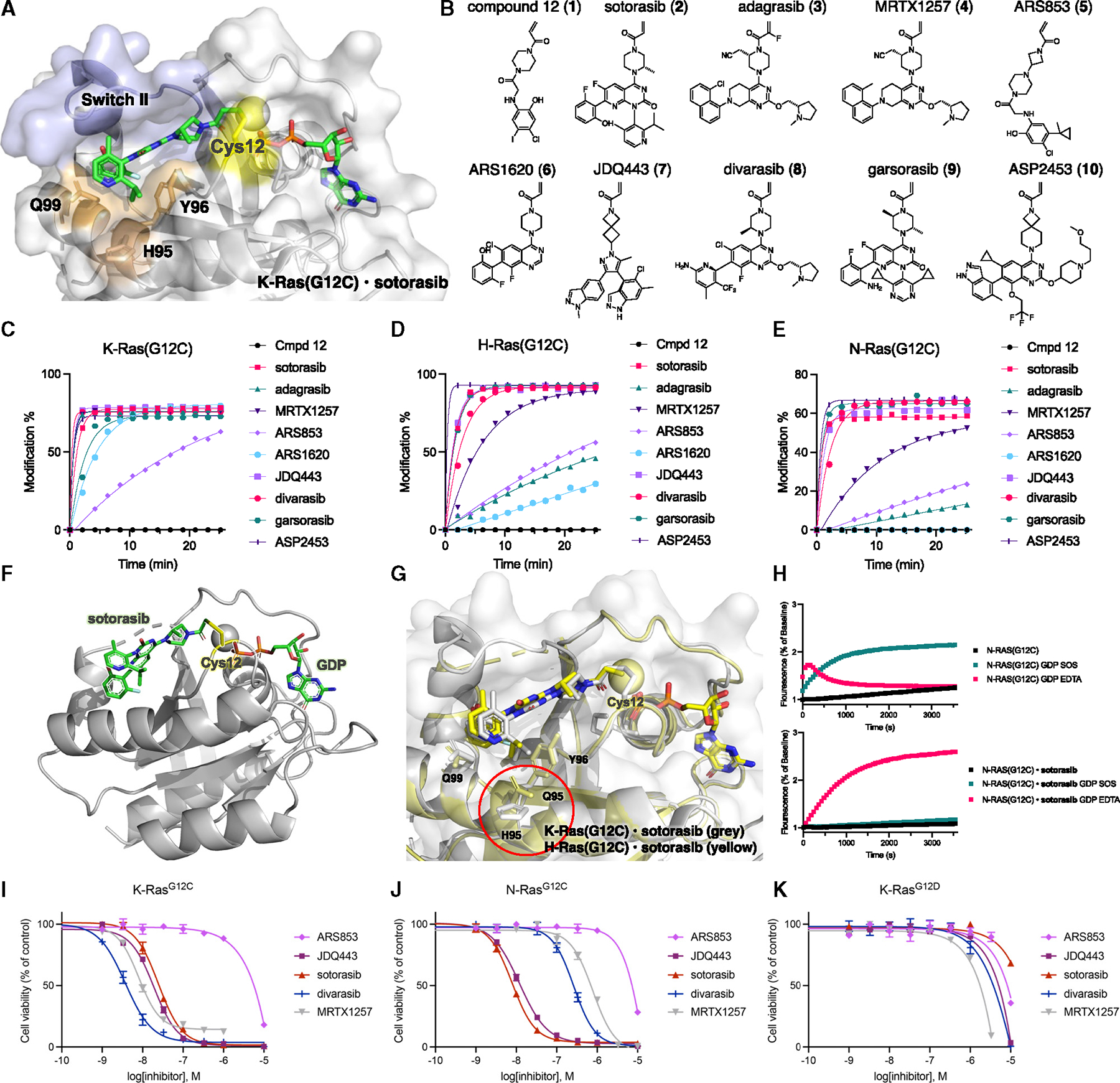
Covalent SII pocket inhibition of K-Ras(G12C), H-Ras(G12C), and N-Ras(G12C) (A) X-ray structure of K-Ras(G12C) bound to sotorasib (PDB: 6OIM). (B) Chemical structures of optimized K-Ras(G12C) inhibitors tested in our screen. (C) Time-dependent covalent modification of K-Ras(G12C) by various compounds (5 μM). (D) Time-dependent covalent modification of H-Ras(G12C) by various compounds (5 μM). (E) Time-dependent covalent modification of N-Ras(G12C) by various compounds (5 μM). (F) Crystal structure of H-Ras(G12C),GDP, sotorasib adduct. (G) Comparison of the structures of K-Ras(G12C),GDP,sotorasib (PDB: 6OIM) and H-Ras(G12C)·GDP·sotorasib (yellow). (H) Intrinsic or SOS- or EDTA-mediated nucleotide exchange of BODIPY-GDP with N-Ras(G12C) 00B7GDP and N-Ras(G12C)·GDP·sotorasib adduct. (I–K) Relative growth of MOLM-13-KRAS-G12C (I), MOLM-13-NRAS-G12C (J), and MOLM-13-KRAS-G12D (K) cells after treatment with K-Ras(G12C) inhibitors for 72 h. Data are presented as mean ± SD (*n* = 3) and are representative of three independent experiments. See also Figure S1.

To study whether other small GTPases also exhibit a targetable SII pocket, we first turned to the K-Ras paralogs H-Ras and N-Ras, which exhibit sequence homology in the SII pocket but differ in position 95 (Q95 in H-Ras and L95 in N-Ras). Both H-Ras and N-Ras can also harbor G12C mutations,^20^ which have been validated as cancer targets in pre-clinical settings.^21,22^ We explored ten different SII pocket inhibitors that have been optimized to target K-Ras(G12C) (Figure 1B) with the respective recombinant protein using mass spectrometry (MS) as a readout for covalent engagement (Figures 1C–1E). We found that some inhibitors were relatively selective for K-Ras(G12C) over H-Ras(G12C) and N-Ras(G12C) (e.g., ARS1620 or adagrasib), while others exhibited very effective labeling of each Ras paralog, with full modification in under 5 min (e.g., sotorasib, JDQ443, ASP2453). To study how the difference in residue 95 affects the binding pose of an SII pocket inhibitor, we solved the crystal structure of sotorasib-bound H-Ras(G12C) (Figure 1F). The Q95 residue is located in closer proximity to the sotorasib binding pocket, which leads to a slight shift in the binding pose closer to the unresolved SII loop (Figure 1G). This suggests that the SII loop remains flexible in the presence of a SII ligand and may be less critical for pocket shape and ligand accommodation compared with residues found on the α3 loop.

To study whether the effective SII pocket target engagement in H-Ras(G12C) and N-Ras(G12C) translates to inhibition and cellular activity, we characterized a small library of 10 compounds that were initially optimized for K-Ras(G12C) in biochemical and cellular settings. Both N-Ras(G12C) and H-Ras(G12C) exhibited markedly increased thermostability in their sotorasib-bound forms, as tested using differential scanning fluorimetry in the presence of SYPRO^™^ Orange (Figures S1A and S1B). To assess functional inhibition, we tested the nucleotide exchange activity of unliganded and sotorasib-bound N-Ras(G12C) (Figure 1H) and H-Ras(G12C) (Figures S1C and S1D) using a fluorescent guanosine diphosphate (GDP) analog (BODIPY-GDP) in the presence of the guanine exchange factor (GEF) son of sevenless (catalytic domain, SOS^Cat^) or ethylenediaminetetraacetic acid (EDTA). Sotorasib inhibited SOS-mediated exchange and significantly reduced the rate of EDTA-mediated exchange in both N-Ras(G12C) and H-Ras(G12C). In the presence of EDTA, unliganded N-Ras(G12C) exhibited a rapid increase in BODIPY-GDP fluorescence, followed by a rapid decrease. This loss of nucleotide is likely due to the compromised stability of N-Ras(G12C) in the presence of EDTA.

To assess the capacity of K-Ras(G12C) drugs to selectively target N-Ras(G12C) in cells, we used acute myeloid leukemia cells that are vulnerable to co-inhibition of FLT3 and Ras paralogs.^23^ This model enabled direct comparison of cellular inhibition of K-Ras(G12C), N-Ras(G12C), and K-Ras(G12D) as a negative control that transforms cells but lacks the targetable cysteine (Figures 1I and 1J). We found that sotorasib (**2**) and JDQ443 (**7**) were equipotent for the cellular inhibition of the Ras paralogs K-Ras(G12C) and N-Ras(G12C), while MRTX1257 (**4**) and divarasib (**8**) exhibited increased cellular potency for inhibition of K-Ras(G12C) over N-Ras(G12C). We also characterized inhibition of pERK and found that sotorasib exhibited increased potency for the inhibition of pERK in N-Ras(G12C) cells over K-Ras(G12C) cells (Figures S1E–S1I). These data suggest that some K-Ras(G12C) inhibitors may be suitable candidates for the treatment of N-Ras(G12C)-driven tumors.

### Targeting of Ras-family GTPases

**Figure 2.**
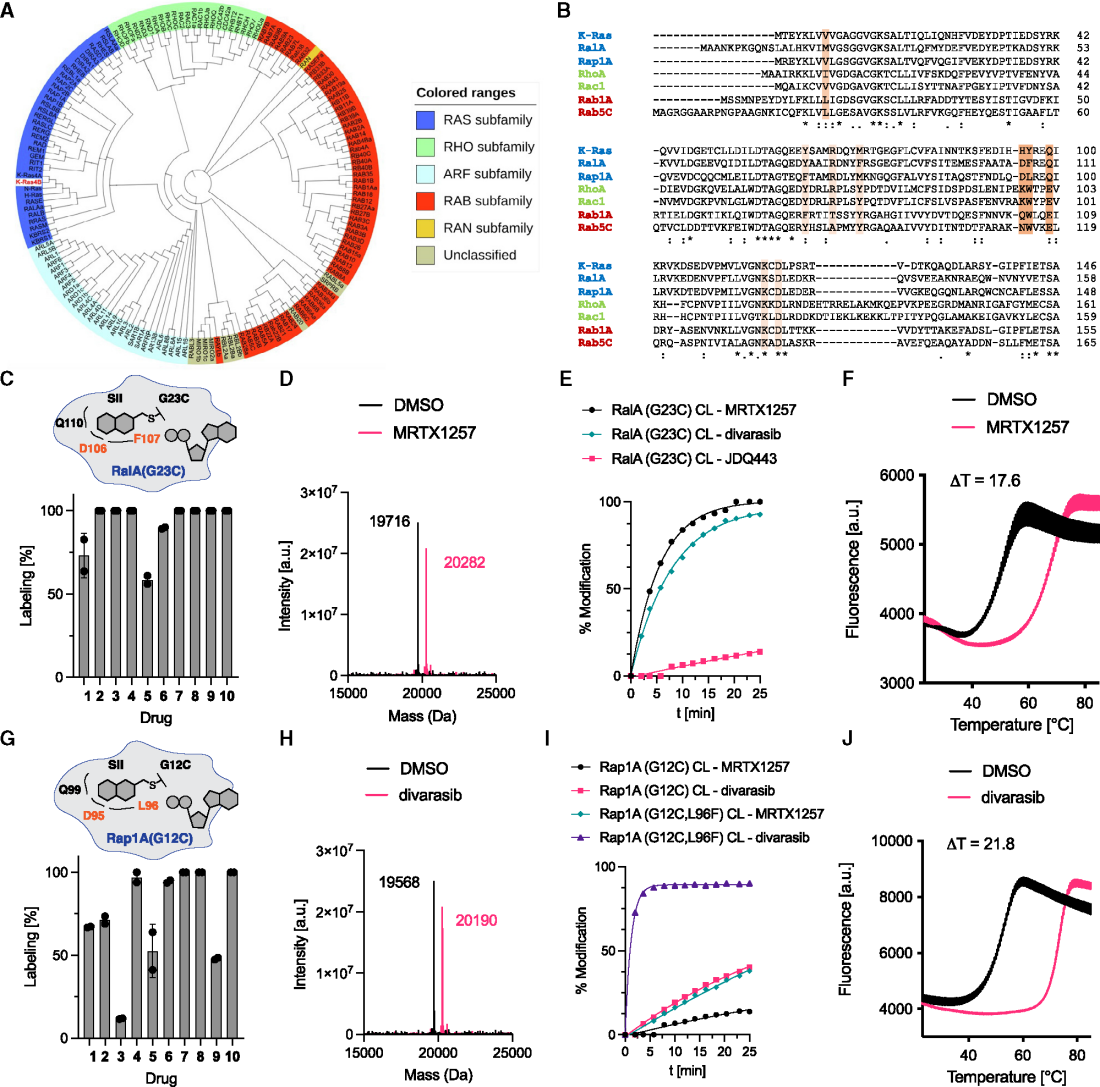
Targeting Ras-family GTPases. (A) Family tree of human superfamily of Ras-like GTPases.^5^ (B) Sequence alignment of various Ras-family GTPases. Residues mediating drug resistance to adagrasib^24^ are highlighted in light orange (rare) and orange (common). (C) Covalent modification of RalA(G23C) with compounds **1–10** (50 μM, 12 h). (D) Intact protein mass spectra of RalA(G23C)·GDP and RalA(G23C)·GDP·MRTX1257 adduct. (E) Time-dependent covalent modification of RalA(G23C) with different compounds (50 μM). (F) Differential scanning fluorimetry of RalA(G23C)·GDP and RalA(G23C)·GDP·divarasib adduct. (G) Covalent modification of Rap1A(G12C) with compounds **1–10** (50 μM, 12 h). (H) Intact protein mass spectra of Rap1A(G12C, L96F)·GDP and Rap1A(G12C,L96F)·GDP·divarasib adduct. (I) Time-dependent covalent modification of Rap1A(G12C) and Rap1A(G12C, L96F) with different compounds (50 μM). (J) Differential scanning fluorimetry of Rap1A(G12C, L96F)·GDP and Rap1A(G12C, L96F)·GDP·divarasib adduct. See also Figure S2.

The Ras superfamily is divided into five main families (Ras, Rho, Rab, Ran, and Arf GTPases) and includes over 150 members (Figure 2A) with varying sequence homology to K-Ras4B (Figure 2B). The Ras family is further divided into six subfamilies (Ras, Ral, Rap, Rad, Rheb, and Rit). RalA (73% homology with K-Ras4B) and Rap1A (74% homology with K-Ras4B) belong to two different subfamilies and exhibit relatively high-sequence homology with K-Ras4B. Both RalA(G23) and Rap1A(G12) feature a glycine in their homologous position to G12 in K-Ras. Unlike the Ras paralogs, these GTPases differ in their residues equivalent to K-Ras(Y96). When we tested the covalent engagement of RalA(G23C) with the different SII pocket inhibitors (Figure 2C), we found full covalent modification with most inhibitors after 12 h. The protein mass after labeling corresponded to the adduct between RalA(G23C) and the respective covalent ligand (Figure 2D is a representative example). Probing the early kinetics of binding revealed that RalA(G23C) exhibited particularly rapid labeling with full engagement after a few minutes with either MRTX1257 or divarasib (Figure 2E). Similar to the Ras paralogs, covalently labeled RalA(G23C) was found to exhibit a marked increase in thermal stability (Figure 2F). Rap1A(G12C), which exhibits similar overall sequence homology, also exhibited full covalent modification with a range of ligands (Figure 2G) corresponding to the desired covalent adducts (Figure 2H is a representative example). Kinetic testing revealed that labeling of Rap1A(G12C) occurred on a slower timescale compared with RalA(G23C), with less than 50% of protein labeled after 25 min (Figure 2I). We speculated that a key difference between these two targets could be the presence of an aromatic amino acid in the 96-equivalent position of RalA(F107) absent in Rap1A(L96). Phenylalanine is structurally closely related to the tyrosine residue found in K-Ras(Y96) and could form π-interactions with the aromatic core of a bound inhibitor. To test this hypothesis, we purified Rap1A(G12C, L96F) and found a marked increase in labeling kinetics (Figure 2I). Strikingly, labeling with divarasib was found to be even faster with this double mutant compared with RalA(G23C) and underwent complete modification in less than 5 min. Correspondingly, a marked shift in protein stabilization was observed with this double mutant (Figure 2J). We tested additional Ras-family GTPases, including M-Ras(G22C), Rit1(G47C), and Rheb(R15C), to assess how generally they could be targeted in this fashion. We found that Rit1(G47C) and M-Ras(G22C) underwent full labeling with several SII pocket inhibitors within 12 h (Figures S2A and S2B), while Rheb(R15C) only underwent partial labeling in this time frame (Figure S2C). M-Ras(G22C) was further found to undergo complete labeling in approximately 10 min with divarasib (Figure S2D). The majority of existing K-Ras drugs are more effective labelers of K-Ras(G12C)·GDP than K-Ras(G12C)·GppNHp, and we expected that this would also translate into more effective targeting of RalA(G23C)·GDP and Rap1A(G12C, L96F)·GDP. To test this, we exchanged GDP with the non-hydrolysable guanosine triphosphate (GTP) analog GppNHp and compared the amount of covalently modified protein after 1 h of labeling (Figure S2E). Both MRTX1257 and divarasib labeled the GDP-bound forms of RalA and Rap1A more effectively than the GppNHp-bound forms.

**Figure 3.**
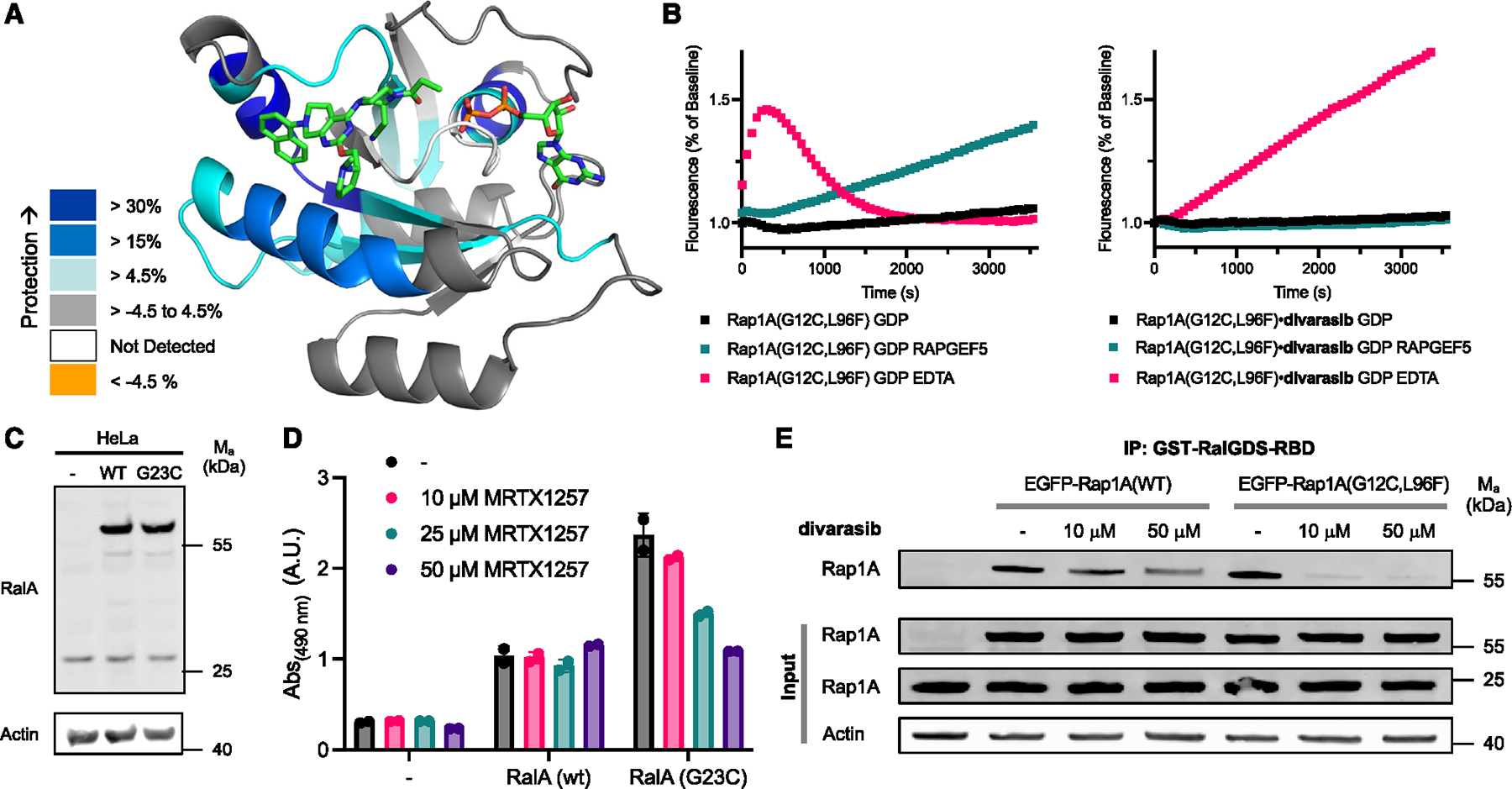
Cellular targeting of Ras-family GTPases. (A) Peptides showing significant differences in HDX at any time point (>0.35 Da and >4.5%) mapped onto a homology model of RalA based on adagrasib-bound K-Ras(G12C) (PDB: 6USZ) according to the legend. (B) Intrinsic or RAPGEF5- or EDTA-mediated nucleotide exchange of BODIPY-GDP with Rap1A(G12C, L96F)·GDP and Rap1A(G12C, L96F)·GDP·divarasib adduct. (C) Immunoblot of HeLa cells transiently overexpressing EGFP-RalA(WT) and EGFP-RalA(G23C). (D) RalA activity measured by RalA G-LISA. HeLa cells were transiently transfected, treated with different concentrations of MRTX1257 for 12 h, and lysates were tested at 0.5 mg/mL. Data are presented as mean ± SEM (n = 2) and are representative of three independent experiments. (E) IP of active GTP-bound Rap1 using GST-RalGDS-RBD of HeLa cells transiently overexpressing EGFP-Rap1A(WT) and EGFP-Rap1A(G12C, L96F) and treated with different concentrations of divarasib. See also Figure S2.

To verify that MRTX1257 binds RalA(G23C) similarly to K-Ras(G12C) in the solution state, we analyzed the adduct by hydrogen-deuterium exchange liquid chromatography-MS (LC-MS) (HDX-MS). This method has been widely applied to analyze the dynamics of SII pocket engagement in K-Ras.^25–28^ HDX-MS analyzes the exchange rate of amide hydrogens, which acts as a surrogate for the stability of secondary structure elements in proteins.^29^ We observed significant decreases in deuterium incorporation throughout large regions of RalA(G23C)·GDP·MRTX1257 adducts relative to RalA(G23C)·GDP. The changes (Figure S2I) were mapped onto the homology model of RalA based on adagrasib-bound K-Ras(G12C) (Figure 3A). Peptides corresponding to the SII (68–74), helix α2 (80–85), and helix α3 (102–112) showed significant protection, but there were also global decreases in exchange throughout large regions of the protein. We found similar results in our previous characterization of SII-pocket-liganded K-Ras mutants.^25,28^ We tested the functional inhibition of the double-mutant Rap1A(G12C, L96F) *in vitro* by adapting our nucleotide exchange assay to a Rap1A GEF, RAPGEF5 (Figure 3B).

We next asked, whether our *in vitro* results could be translated into cellular inhibition of RalA(G23C) and Rap1A(G12C, L96F). To this end, we transiently overexpressed EGFP-tagged versions of RalA(wild-type [WT]) and RalA(G23C) (Figure 3C) and tested their cellular activity using a RalA G-LISA assay based on the capture of active GTP-bound RalA (Figure 3D). Transient overexpression of RalA(WT) increased the amount of active RalA but was found to be insensitive to increasing concentrations of MRTX1257. Overexpression of RalA(G23C) further elevated the amount of active RalA, which suggests that this mutant isoform is dominant-active. Upon incubation with MRTX1257, the activity of RalA(G23C) decreased in a dose-dependent fashion, indicative of cellular inhibition of this GTPase. To assay cellular inhibition of Rap1A(G12C, L96F), we affinity-purified GTP-bound active Rap1A using GST-RalGDS-RBD and subsequently immunoblotted bound and unbound fractions (Figure 3E). Treatment of cells with divarasib effectively inhibited active Rap1A(G12C, L96F). Next, we tested whether divarasib exhibits reversible binding affinity to RalA and Rap1A. As such, this compound could present a valuable starting point for the development of reversible GTPase inhibitors that do not require the introduction of a covalent handle. To this end, we conducted surface plasmon resonance measurements with RalA(WT), Rap1A(WT), and RhoA(WT). We could detect reversible binding of divarasib to both RalA(WT) and Rap1A(WT) but not to RhoA(WT), which exhibits less sequence homology with K-Ras and was included as a potential negative control (Figures S2F–S2H). These data suggest that the development of reversible inhibitors for GTPases beyond the Ras paralogs could be feasible.

### Cellular engagement of Ras-family GTPases with RMC-6291

**Figure 4.**
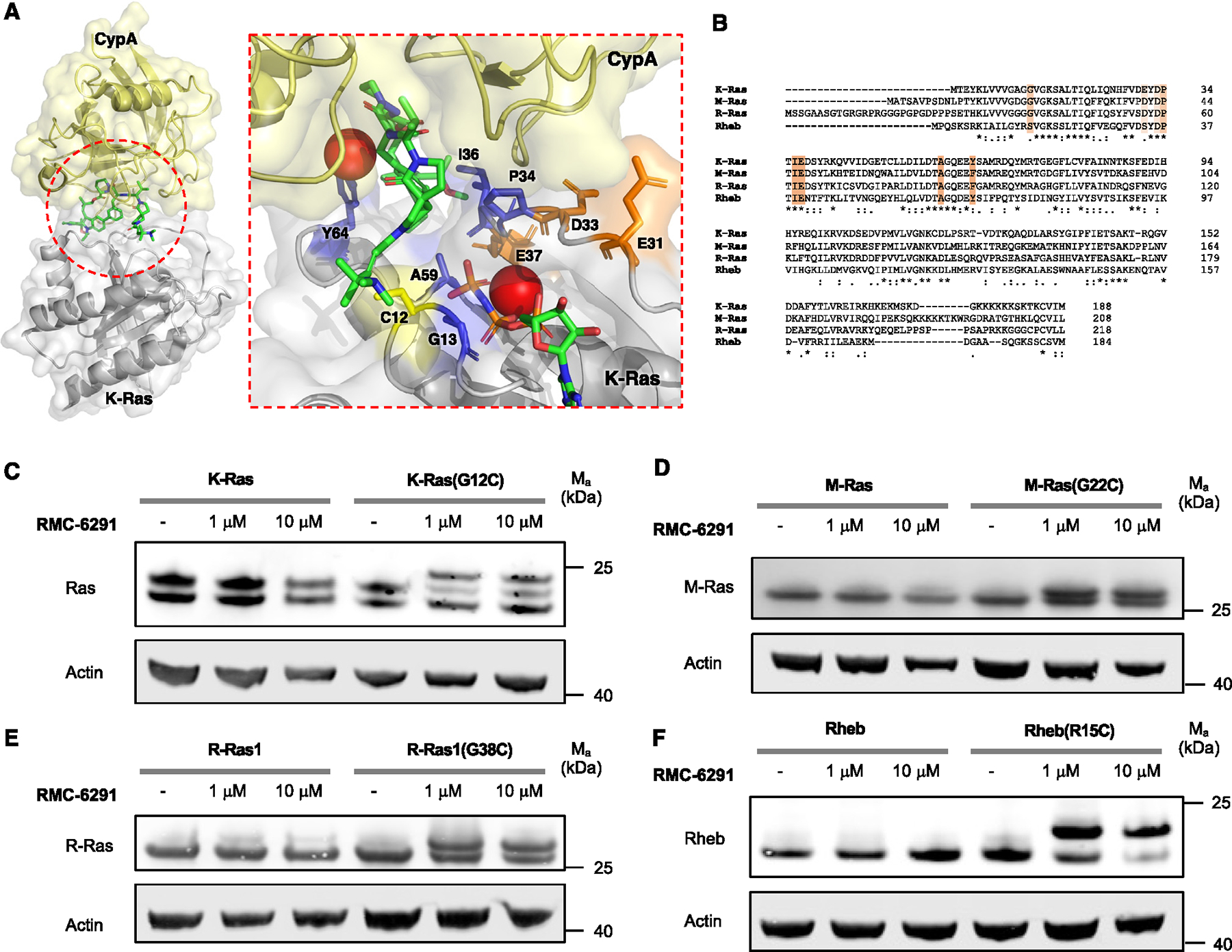
Cellular targeting of Ras-family GTPases with RMC6291. (A) X-ray structure of tricomplex of RMC-4998, K-Ras(G12C), and CypA (PDB: 8G9P). K-Ras residues involved in binding RMC-4998 are colored in blue. Negatively charged K-Ras residues involved in binding CypA are colored in orange. (B) Sequence alignment of Ras-family GTPases K-Ras, M-Ras, R-Ras, and Rheb. Residues mediating drug resistance to RMC-4998 are highlighted in light orange (mild effect) and orange (strong effect). (C) Immunoblot of HeLa cells transiently overexpressing K-Ras or K-Ras(G12C). HeLa cells were transiently transfected, treated with different concentration ofRMC-6291 for 3 h, and blotted for Ras. (D) Immunoblot of HeLa cells transiently overexpressing M-Ras or M-Ras(G22C). HeLa cells were transiently transfected, treated with different concentration ofRMC-6291 for 3 h, and blotted for M-Ras. (E) Immunoblot of HeLa cells transiently overexpressing R-Ras1 or R-Ras1(G38C). HeLa cells were transiently transfected, treated with different concentration ofRMC-6291 for 3 h, and blotted for R-Ras. (F) Immunoblot of HeLa cells transiently overexpressing Rheb or Rheb(R15C). HeLa cells were transiently transfected, treated with different concentration of RMC-6291 for 3 h, and blotted for Rheb. Data are representative of three independent experiments.

In addition to targeting K-Ras(G12C) with structurally related SII pocket inhibitors, such as compounds **1–10**, an alternative strategy has recently been reported that labels K-Ras(G12C) based on the formation of a tricomplex between K-Ras(G12C), a chemical ligand, and the immunophilin cyclophilin A (CypA). As the tricomplex inhibitor strategy relies on different molecular recognition determinants from SIIP binders, including recognition of enhanced binding to the GTP state, we asked whether this strategy could expand the chemical matter applicable to chemical targeting of GTPases with an introduced cysteine. Extensive characterization of an early-optimized ligand, RMC-4998, was recently reported, including a crystal structure of K-Ras(G12C):RMC-4998:CypA (PDB: 8G9P).^30^ The K-Ras residues most critical for the formation of this tricomplex were found to be C12, G13, E31, D33, P34, I36, E37, A59, and Y64. The negatively charged residues E31, D33, and E37 were found to form hydrogen bonds with residues on CypA, while the other residues were found to be important for facilitating ligand binding (Figure 4A). Notably, most of these residues are orthogonal to ones involved in the binding of SII ligands explored above and could therefore provide an attractive alternative approach to target Ras-family GTPases. We sought to test opportunities for cellular engagement of Ras-family GTPases with high-sequence homology in the residues listed and identified M-Ras, R-Ras1, and Rheb. M-Ras and R-Ras1 differ in their E31- and Y64-equivalent residues, and both exhibit an aspartic acid in the 31-equivalent and a phenylalanine in the 64-equivalent position. Rheb differs in the G13- and E31-equivalent residues and exhibits a serine in both positions (Figure 4B). We tested cellular engagement by transiently overexpressing the respective GTPase in WT form or as G12C-equivalent point mutant, treated with the clinical candidate RMC-6291, and blotted for the respective GTPase. For all four of the tested GTPases, we found a band shift for the G12C-mutant form in the presence of an inhibitor, consistent with the formation of a covalent complex with decreased gel mobility (Figures 4C–4F). This demonstrates that the tricomplex inhibitor RMC-6291 also enables targeting of GTPases beyond K-Ras and could be an attractive alternative to other SII pocket ligands characterized in this study. When we tested mutants of Rab and Rho GTPases (Rac1(G12C) and Rab1A(S20C)), we could not detect a band shift. This is consistent with the lower sequence homology between the residues critical for interaction with CypA and ligand engagement.

### Targeting of Rho- and Rab-family GTPases

**Figure 5.**
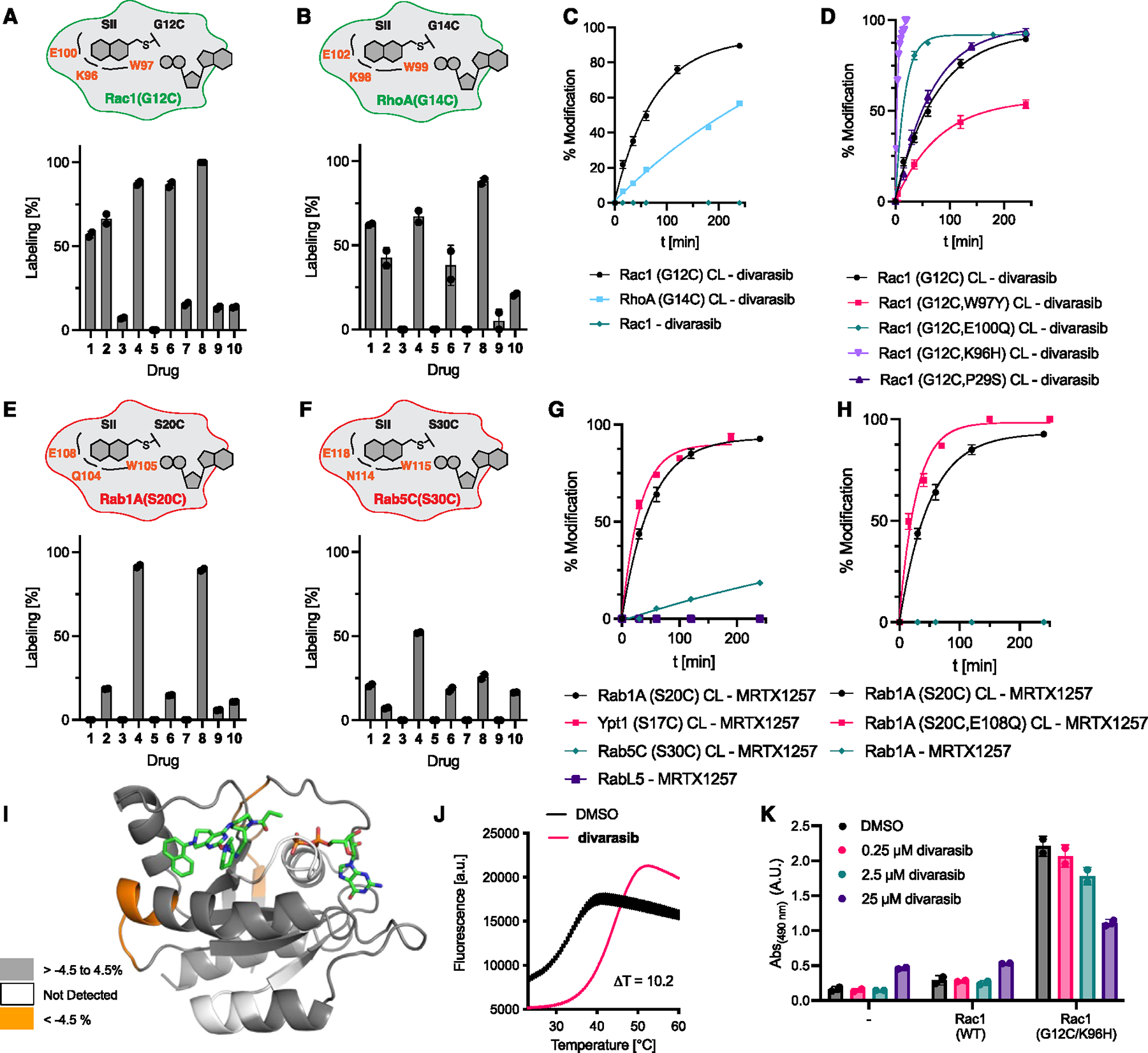
Targeting Rho- and Rab-family GTPases. (A) Covalent modification of Rac1(G12C) with compounds **1–10** (50 μM, 12 h). (B) Covalent modification of RhoA(G14C) with compounds **1–10** (50 μM, 12 h). (C) Time-dependent covalent modification of Rac1(G12C), RhoA(G14C), and Rac1(WT) with divarasib (50 μM). (D) Time-dependent covalent modification of various Rac1 mutants with divarasib (50 μM). (E) Covalent modification of Rab1A(S20C) with compounds **1–10** (50 μM, 12 h). (F) Covalent modification of Rab5C(S30C) with compounds **1–10** (50 μM, 12 h). (G) Time-dependent covalent modification of Rab1A(S20C), Ypt1(S17C), Rab5C(S30C), and RabL5(WT) with MRTX1257 (50 μM). (H) Time-dependent covalent modification of various Rab1A mutants with MRTX1257 (50 μM). (I) Peptides showing significant differences in HDX at any time point (>0.35 Da and >4.5%) mapped onto a homology model of Rab1A based on adagrasib-bound K-Ras(G12C) (PDB: 6USZ). (J) Differential scanning fluorimetry of Rac1(G12C, K96H)·GDP and Rac1(G12C, K96H)·GDP·divarasib adduct. (K) Rac1 activity measured by Rac1 G-LISA. HeLa cells were transiently transfected, treated with different concentrations of divarasib for 12 h, and lysates were tested at 0.5 mg/mL. Data are presented as mean ± SEM (*n* = 2) and are representative of three independent experiments. See also Figures S2, S3, and S4.

To test the potential for targeting Rho- and Rab-family GTPases through SII pocket engagement, we recombinantly expressed several widely studied members of these families. For Rho GTPases, we chose RhoA and Rac1, and for Rab GTPases, we selected Rab1A and Rab5C. Interestingly, both the K-Ras(Y96)- and K-Ras(Q99)-equivalent positions are highly conserved across Rho and Rab GTPases, suggesting that our results on the selected examples could be representative and generalizable for other members of these families. Most Rho and Rab GTPases contain a tryptophan in the position equivalent to K-Ras(Y96) and glutamic acid in the position equivalent to K-Ras(Q99). Our MS-based drug screen revealed that Rac1(G12C) and RhoA(G14C) were effectively labeled by MRTX1257 and divarasib (Figures 5A and 5B). In contrast to the Ras-family GTPases, fewer drugs designed to target K-Ras (G12C) were capable of effectively labeling Rho- and Rab-family members, which could be an effect of the described variations in the α3 helix residues in this family compared with the Ras subfamily. Rac1(G12C) was fully modified with divarasib within 240 min (Figure 5C). Permutations of residues in the SII pocket revealed that Y96 is less well matched with the Rac1 SII pocket than W96, suggesting that all three aromatic amino acids (Y, F [e.g., RalA], and W) are well tolerated in this position (Figure 5D). Introducing Q100 into Rac1(G12C) yielded the double-mutant Rac1(G12C, E100Q), which significantly accelerated covalent modification. Introducing H96 yielded the double-mutant Rac1(G12C, K96H), which had an even more pronounced effect and enabled modification of the Rac1 SII pockets within minutes and on similar timescales to Ras-family GTPases RalA(G23C), Rap1A(G12C, L96F), and M-Ras(G22C). The large favorable impact on binding of this mutation was unexpected because we had previously established that various residues can be tolerated in this position equivalent to K-Ras(H95), e.g., in N-Ras, H-Ras, or other Ras GTPases. This finding reinforced the importance and potential of this residue as a selectivity filter in the design of SII pocket inhibitors. Finally, we decided to test Rac1(G12C, P29S) because P29S is one of the most common cancer-activating mutations occurring in Rac1 and found that this mutation had little impact on SII pocket labeling (Figure 5D).

To study the stabilization divarasib labeling affords to Rac1(G12C), we conducted a differential scanning fluorimetry experiment and found that Rac1(G12C)·GDP·divarasib did not exhibit increased temperature stability compared with Rac1(G12C)·GDP (Figure S3A). To test the nucleotide-dependence of labeling, we exchanged nucleotides in Rac1(G12C, K96H) and measured labeling of nucleotide-free, GDP-loaded, and GTPγS-loaded protein in parallel (Figures S3B and S3C). We found that GTPase loaded with the GTP analog GTPγS could undergo covalent labeling with divarasib, though it was incomplete. In its nucleotide-free state, divarasib did not effectively label the GTPase.

Similar to the tested Rho GTPases, MRTX1257 and divarasib were also the most effective covalent ligands for the Rab GTPases Rab1A(S20C) and Rab5C(S30C) (Figures 5E and 5F). Although Rab1A(S20C) and its yeast homolog Ypt1(S17C) underwent full labeling with MRTX1257 in 240 min, Rab5C(S30C) was less effectively targeted and was only partially labeled in this time frame (Figure 5G). We also tested RabL5 and SRPRB, which are, to the best of our knowledge, the only GTPases with native cysteines at the position equivalent to K-Ras(G12) and could therefore be a potentially concerning off-target for chemical genetics experiments. RabL5 (unclassified, low-sequence homology to Rab GTPases) and signal recognition particle subunit beta (SRPRB—Figure S2D) did not undergo detectable covalent modification within 3 h, indicating that our strategy would enable selective targeting of desired GTPases without background labeling of endogenous GTPases. Finally, we tested Rab1A(S20C, E108Q) to study whether the introduction of a second mutation in the SII pocket could enhance binding similarly to the Rho GTPases. Indeed, Rab1A(S20C, E108Q) exhibited faster labeling kinetics than Rab1A(S20C), and Rab1A(WT) did not undergo labeling (Figure 5H). We analyzed the Rab1A(S20C, E108Q),GDP,MRTX1257 adduct by HDX LC-MS (HDX-MS) and found increased exchange induced by the compound in residues in the vicinity of the SII pocket at positions 48 and 112 (Figures 5I and S2J). This result stands in contrast with the protection observed in the HDX-MS experiment with RalA(G23C). Together, our results suggest that the K-Ras(G12C)-optimized ligands tested herein are less well matched to target the SII pockets of the Rho and Rab GTPases tested compared with members of the Ras family and need to be optimized through medicinal chemistry efforts. Alternatively, a secondary mutation could be introduced to accelerate binding as seen with the double-mutant Rac1(G12C, K96H). To further assess whether this secondary mutation enhances binding and enables targeting of a Rho GTPase in cells, we conducted differential scanning fluorimetry experiments and measured Rac1 activity in cells. We found that the effective *in vitro* labeling of this double mutant translated into significant stabilization (10.2°C) of Rac1(G12C, K96H)·GDP·divarasib over Rac1(G12C, K96H)·GDP (Figure 5J). In HeLa cells transiently expressing GFP-Rac1(G12C, K96H), divarasib afforded dose-dependent inhibition of Rac1 activity. Cellular engagement of GFP-Rac1(G12C, K96H) was verified via targeted proteomics,^31^ where a covalently modified Rac1 peptide was detected after cellular treatment with divarasib (Figures S3D–S3F). To characterize the signaling effects of Rac1 mutant inhibition, we utilized COS7 cells, an established immortalized cell line commonly used to demonstrate the signaling effects of Rac1 mutant overexpression.^32^ Additionally, we included the clinically relevant mutant Rac1 (P29S) as a positive control for Rac1 oncogenic signaling, as well as the Rac1 (G12C, P29S, K96H) triple mutant to examine the effects of covalent modification of the SII pocket on this phenotype. As shown in the PAK1 IP experiment, cells overexpressing Rac1 (G12C) and/or (P29S) mutations showed increased Rac1 interaction with PAK1 in comparison with Rac1 (WT). Furthermore, both the Rac1 (G12C, K96H) and Rac1 (G12C, P29S, K96H) exhibited a strong reduction in pull-down after drug treatment, indicating that covalent engagement of divarasib inhibits Rac1 binding to PAK1(Figure S4A). To further characterize the effects of divarasib on downstream signaling of Rac1 G12C mutants, we performed a western blot analysis in COS7 cells treated in low-serum conditions. All cells transiently expressing Rac1 (G12C) and (P29S) mutations showed increased phosphorylation of cofilin, which promotes tumor cell migration via regulating actin cytoskeleton organization. In cells transiently expressing Rac1(G12C, P29S, K96H), we reproducibly saw partial inhibition of p-cofilin after treatment with divarasib (Figure S4B).

### Development of optimized inhibitor scaffolds

**Figure 6.**
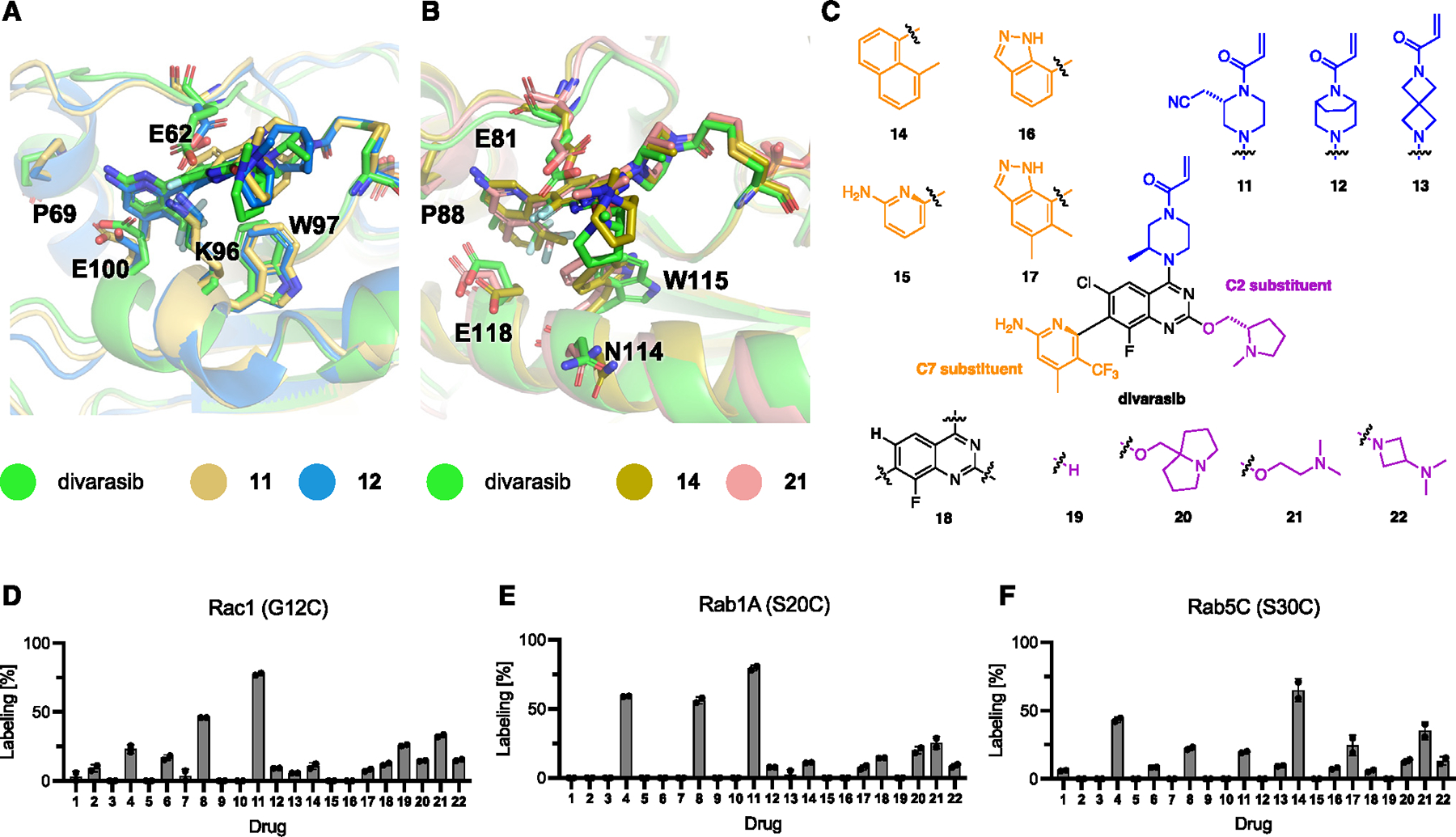
Ligand optimization for Rab and Rho GTPases. (A and B) Representative binding poses from covalent MD simulations of divarasib, and selected ligands in Rac1(G12C) (A) and Rab5C(S30C) (B), respectively. (C) Chemical structures of novel SII pocket inhibitors to improve targeting of Rab and Rho GTPases. (D) Covalent modification of Rac1(G12C) with compounds **1–22** (50 μM, 1 h). (E) Covalent modification of Rab1A(S20C) with compounds **1–22** (50 μM, 1 h). (F) Covalent modification of Rab5C(S30C) with compounds **1–22** (50 μM, 12 h). See also Figures S5 and S6.

Although the introduction of a secondary mutation enhances binding and could be an attractive strategy for chemical genetics experiments with GTPases, differences in the SII pocket residues between GTPases suggests great potential for the development of optimized and selective ligands for various GTPases. While the optimization of refined chemical leads for various GTPases is beyond the scope of this initial discovery study, we sought to explore novel chemical space for Rac1(G12C), Rab1A(S20C), and Rab5C(S30C). To account for the highly dynamic nature of the SII pocket, we used molecular dynamics (MD) simulations to predict the optimal binding pose for novel analogs of MRTX1257 and divarasib and applied quantum mechanical (QM) calculations to understand the reactivities of chemical warheads (Figures S5A–S5D). We designed ligands to maximize interactions within the binding pockets and predicted the lowest energy conformation for each ligand. At the same time, we observed major differences between the SII pockets of Rac1 and the two studied Rab GTPases, Rab1A and Rab5C, which could enable selective targeting (Figures 6A and 6B). To explore potential new ligands beyond those optimized against K-Ras (G12C), we designed new configurations of SII pocket covalent binders. Our extended library of 22 compounds included modifications of multiple constituents of the divarasib ligand (Figure 6C). The total set of screening results from the new compounds **11–22** and the set of initially screened ligands enabled the identification of ligands that exhibited more effective covalent labeling with each of the tested GTPases (Figures 6D–6F). Compound **11** underwent faster labeling of Rac1(G12C) and Rab1A(S20C), and compound **14** underwent faster labeling of Rab5C(S30C).

Combining computational modeling and testing of new analogs, we developed a better understanding of the structure-activity relationships (SARs) for SII pocket ligands outside of the K-Ras family that can guide further optimization: in Rac1(G12C) screening, compounds **8** and **11** exhibited the fastest labeling rates, suggesting that the 2-amino-4-methyl-5-trifluoromethyl-pyridine moiety is the preferred C7-substituent for the reversible binding into Rac1 SII pocket (Figure 6D). We measured the labeling kinetics and found that compound **11** was able to label Rac1(G12C) approximately 2-fold faster than divarasib (**8**) (Figure S5E). Alternate positioning in the pocket and/or increased warhead reactivity could contribute to this faster labeling. Compounds **8, 11, 12,** and **13** exhibited nearly identical SII pocket binding poses in Rac1(G12C) modeling; however, the different intrinsic warhead reactivities (**8, 11, and 12**) and non-optimal warhead positioning (**13**) may explain the varying level of cysteine labeling observed. However, Rab1A(S20C) screening did not clearly show additive effects from the C7-substituents and warhead (Figure 6E). For example, two out of three compounds among **4, 8,** and **11** either share the same C7-substituent or the same warhead; but all underwent faster covalent labeling than other compounds. For Rac5C(S30C), methylnaphthalene was the optimal C7-substituent (**4** and **14**),leading to the fastest labeling rates, possibly through better reversible binding affinity as suggested by modeling (Figure 6F). At the same time, very different SARs were observed for the C2-position between these three GTPases. First, a relatively flexible C2-substituent (**21**) outperformed two relatively rigid C2-substituents (**20** and **22**). Second, a hydrogen at the C2-position (in **6** and **19**) was relatively well tolerated in Rac1(G12C) but decreased the labeling rate in Rac1A(G20C) and Rac5C(S30C). We hypothesized that the reduced impact of substituents in the C2-position of Rac1(G12C) could be due to a lysine in position 96 (equivalent with K-Ras(H95)), which could lead to significant steric clash around the C2-position. We tested our extended library for labeling of K-Ras(G12C) and found that all compounds still exhibited fast labeling of K-Ras(G12C) (Figure S5F). Combined, these results demonstrate an initial optimization of the K-Ras(G12C)-optimized ligands divarasib and MRTX1257 to target Rab and Rho GTPases and provide design considerations for the next generation of optimized ligands targeting these proteins selectively.

### Concluding remarks

Here, we demonstrate that a targetable cryptic SII pocket exists in many GTPases beyond K-Ras. Cysteine-mutants of different Ras-, Rho-, and Rab-family GTPases undergo fast and selective covalent modification with SII pocket inhibitors initially optimized for K-Ras(G12C). Targeting the Ras paralogs H-Ras(G12C) and N-Ras(G12C) was highly effective and could provide immediate potential for clinical translation. Targeting the Ras GTPases RalA(G23C) and Rap1A(G12C, L96F) was effective *in vitro* and in cells demonstrating the utility of SII pocket inhibitors for other Ras-subfamilies.

Several Ras GTPases, including M-Ras(G22C), R-Ras1(G38C), and Rheb(R15C), could be targeted in cells using the chemically distinct macrocycle RMC-6291, which makes interactions with the GTPase that are orthogonal to that of other SII pocket inhibitors. As Rheb(R15C) was only modestly targetable by SII pocket ligands, but was efficiently targeted by RMC-6291, the tricomplex strategy has the potential to expand targeting across the GTPase family.

Targeting the Rho GTPases Rac1(G12C) and RhoA(G14C) and the Rab GTPase Rab1A(S20C) was effective *in vitro*, but inhibitors optimized for K-Ras(G12C) were less well matched to target their SII pockets. The introduction of a second mutation to enhance binding of existing inhibitors in Rac1(G12C, K96H) could overcome this limitation and enabled effective targeting in cells. In parallel, we demonstrated the development of a first set of optimized inhibitors for Rac1(G12C), Rab1A(S20C), and Rab5C(S30C), suggesting great potential for future medicinal chemistry efforts to target SII pockets in other GTPases more effectively and selectively.

Some of the inhibitors initially optimized to target K-Ras (MRTX1257 or divarasib) enabled the targeting of various GTPases, while others (e.g., JDQ443 or ASP2453) were highly selective for K/H/N-Ras, demonstrating potential for the development of both promiscuous and selective SII-pocket-binding ligands. Through structural analysis, sequence alignments, and whole protein MS to assess covalent engagement, we identified residues that are particularly critical for SII pocket engagement and criteria for which amino acids are tolerated in these positions. For example, various aromatic amino acids are well tolerated in the K-Ras(Y96) equivalent position and development of Rho and Rab GTPase-selective inhibitors can benefit from the presence of a conserved tryptophan in this position. Various residues are tolerated in the K-Ras(H95) equivalent position, but they can serve as a selectivity filter, and H95-mediated ligand interaction can significantly enhance the binding of inhibitors, though some SII pocket scaffolds are less dependent on this interaction.^13^

The ability to covalently target engineered cysteine-containing mutants of other GTPases bears great potential for chemical genetics approaches to studying GTPases selectively in a proteome. For those interested in GTPases not studied explicitly here, we include a flow chart for sequence analysis and matching of candidate inhibitors based on sequence alignment of any small GTPase family member to K-Ras (Figure S6). Clinical translation of our findings to GTPases beyond K/H/N-Ras will require further compound optimization to design selective and reversible inhibitors. The feasibility of reversible SII pocket targeting was recently demonstrated with MRTX1133 targeting K-Ras(G12D).^33^ Although a glycine in K-Ras(G12) equivalent position is most common, some small GTPases feature a native serine (Rab GTPases) or arginine (e.g., Rheb) in this position, allowing us to explore targeting these with covalent chemistry recently developed for the equivalent K-Ras mutant alleles K-Ras(G12S),^34^ K-Ras(G12R),^35^ and K-Ras(G12D).^36^

### Limitations of the study

Demonstrating the targetability of multiple classes of GTPases presents a significant advance in the field and opens numerous opportunities for chemical genetics, probe development, and drug discovery. However, the present study is still biased toward chemical space optimized to target K-Ras(G12C). New chemical space and the discovery of novel types of reversible high-affinity scaffolds for other GTPases requires a concerted medicinal chemistry campaign that lies beyond the scope of this initial study.

## RESOURCE AVAILABILITY

### Lead contact

Further information and requests for resources and reagents should be directed to and will be fulfilled by the lead contact, Kevan M. Shokat (kevan.shokat@ucsf.edu)

### Materials availability

Key plasmids generated in this study will be deposited to Addgene upon publication. Addgene IDs are available in the key resources table. Compounds generated in this study are available through custom synthesis from Shanghai Medicilon Inc. MOLM-13 cell lines generated and used in this study are available from author Benjamin J. Huang (Ben.Huang@ucsf.edu).

### Data and code availability

- X-ray crystallography data has been deposited to PDB and is publicly available as of the date of publication. The accession code is specified in the key resources table.
- The MS proteomics data have been deposited to the ProteomeXchange Consortium through the PRIDE partner repository with the dataset identifiers PXD041440 and PXD054414.
- This paper does not report original code.
- Any additional information required to reanalyze the date reported in this paper is available from the lead contact upon request.

## STAR★METHODS

### EXPERIMENTAL MODEL AND STUDY PARTICIPANT DETAILS

#### Cell culture

HeLa cells were culture at 37°C, 5% CO_2_ in DMEM (Thermo Fisher) supplemented with 10% heat-inactivated FBS (HyClone) and 1% Penicillin-Streptomycin (Thermo Fisher). Early passage MOLM-13 cells (DSMZ) were cultured at 37°C, 5% CO_2_ in RPMI media (HyClone) containing 10% fetal bovine serum (Corning), 1% penicillin-streptomycin (Thermo Fisher), and 1% GlutaMAX (Thermo Fisher). Cos7 cells were culture at 37°C, 5% CO_2_ in DMEM (Thermo Fisher) supplemented with 10% heat-inactivated FBS (HyClone) and 1% Penicillin-Streptomycin (Thermo Fisher).

### METHOD DETAILS

#### Recombinant protein expression and purification

DNA sequences encoding human GTPases Cyslight (all C→S) were codon optimized, synthesized, and cloned into the pProEx vector by Genscript. The resulting construct contains N-terminal 6xHis tag and a TEV cleavage site (ENLYFQG). The proteins were expressed and purified following previously reported protocols.^12,41^ Briefly, chemically competent BL21(DE3) cells were transformed with the corresponding plasmid and grown on LB agar plates containing 50 μg/mL carbenicillin. A single colony was used to inoculate a culture at 37°C, 220 rpm in terrific broth containing 50 μg/mL carbenicillin. When the optical density reached 0.6, the culture temperature was reduced to 18°C, and protein expression was induced by the addition of IPTG to 1 mM. After 16 h at 18°C, the cells were pelleted by centrifugation (6,500 × g, 10 min) and lysed in lysis buffer [20 mM Tris 8.0, 500 mM NaCl, 5 mM imidazole] with EDTA-free protease inhibitor using ultrasonication. The lysate was clarified by high-speed centrifugation (19,000 × g, 15 min), and the supernatant was used in subsequent purification by immobilized metal affinity chromatography (IMAC). His-TEV tagged protein was captured with Co-TALON resin (Clonetech, Takara Bio USA, 2 mL slurry/liter culture) at 4°C for 1 h with constant end-to-end mixing. The loaded beads were then washed with lysis buffer (50 mL/liter culture), and the protein was eluted with elution buffer [20 mM Tris 8.0, 300 mM NaCl, 300 mM imidazole]. To this protein solution was added His-tagged TEV protease (0.05 mg TEV/mg Ras protein) and GDP (1 mg/mg Ras protein), and the mixture was dialyzed against TEV Cleavage Buffer [20 mM Tris 8.0, 300 mM NaCl, 1 mM EDTA, 1 mM DTT] at 4°C using a 10K MWCO dialysis cassette until LC-MS analysis showed full cleavage (typically 16–24 h). MgCl_2_ was added to a final concentration of 5 mM, and the mixture was incubated with 1 mL Ni-NTA (Qiagen) beads at 4°C for 1 h to remove TEV protease and any residual His-tagged proteins and peptides. The protein solution was diluted 1:10 v/v with 20 mM Tris 8.0 and further purified with anion exchange chromatography (HiTrapQ column, GE Healthcare Life Sciences) using a NaCl gradient of 50 mM to 500 mM in 20 mM Tris 8.0. For some GTPases, the anion exchange column was omitted. The protein was concentrated using a 10K MWCO centrifugal concentrator (Amicon-15, Millipore) to 20 mg/mL and purified by size exclusion chromatography on a Superdex 75 10/300 GL column (GE Healthcare Life Sciences). Fractions containing pure protein were pooled and concentrated to 20 mg/mL, flash frozen with liquid nitrogen, and stored at −78°C.

#### GppNHp-loaded protein method

30 μL 100 mM EDTA in HEPES buffer (10 mM final) and 30 μL 50 mM GppNHp in HEPES buffer (10 mM final) were added to 240 μL of 5 μM purified GTPase in HEPES buffer and incubated for 10 min at room temperature. 5 μL 2 M MgCl_2_ solution was added to quench the reaction. The GppNHp loaded GTPase was purified using a Zeba 7kDa MWCO desalting spin column and used for intact protein mass spectrometry.

#### Intact protein mass spectrometry

Purified GTPase variants (1 μM final) were incubated with compounds at 50 μM (1% v/v DMSO final) in 20 mM HEPES pH 7.5, 150 mM NaCl, 1 mM MgCl2 in a total volume of 150 μL. After the noted time, the samples were analyzed by intact protein LC/MS using a Waters Xevo G2-XS system equipped with an Acquity UPLC BEH C4 1.7 μm column. The mobile phase was a linear gradient of 5–95% acetonitrile / water + 0.05% formic acid. The spectra were processed using QuantLynx, giving the ion counts observed for the most abundant species.

#### Differential scanning fluorimetry

The protein of interest was diluted with HEPES Buffer [20 mM HEPES 7.5, 150 mM NaCl, 1 mM MgCl_2_] to 2 μM. 1 μL of SYPRO Orange (500x) was mixed with 99 μL of protein solution. This solution was dispensed into wells of a white 96-well PCR plate in triplicate (25 μL/well). Fluorescence was measured at 0.5C temperature intervals every 30 s from 25°C to 95°C on a Bio-Rad CFX96 qPCR system using the FRET setting. Each data set was normalized to the highest fluorescence and the normalized fluorescence reading was plotted against temperature in GraphPad Prism 8.0. Tm values were determined as the temperature(s) corresponding to the maximum of the first derivative of the curve.

#### GEF- or EDTA-mediated nucleotide exchange

This assay was performed as previously reported^12,42–44^ with slight modifications. To 25 μL of a 10 μM solution of GDP-loaded GTPase or compound-labeled GDP-loaded GTPase in HEPES buffer was added 175 μl of a 1 μM BODIPY-GDP (Thermo Scientific G22360) solution in HEPES buffer (final concentrations 1.25 μM GTPase and 1.0 μM BODIPY-GDP). 12 μL of this solution (triplicate for each condition) was added to wells of a black 384-well low-volume assay plate (Corning 4514). 3 μL of either HEPES, 5 μM GEF, or 40 mM EDTA (all prepared in HEPES Buffer) were added via a multichannel pipet rapidly to the wells. This should take less than 15 s to finish. The plate was immediately placed in a TECAN Spark 20M plate reader, and fluorescence for BODIPY (excitation 488 nm, emission 520 nm) was read every 60 s over 1 h. Fluorescence intensity was normalized to values at time 0 and plotted against time.

#### Crystallization and structure determination

GTPase Cyslight bound by GDP and purified by size exclusion chromatography was diluted to 100 μM in Reaction Buffer (20 mM HEPES 7.5, 150 mM NaCl, 1 mM MgCl_2_). Compound was added as a 10 mM solution in DMSO to a final concentration of 200 μM. The mixture was allowed to stand at 23°C until LC-MS analysis of the reaction mixture showed full conversion to a single covalent adduct. The reaction mixture was purified by size exclusion chromatography (Superdex75, 20 mM HEPES 7.5, 150 mM NaCl, 1 mM MgCl_2_) and concentrated to 20 mg/mL. Crystals were grown at 20°C in a 96-well plate using the hanging-drop vapor diffusion method. The crystals were transferred to a cryoprotectant solution (0.1 M MES pH 6.5, 30% w/v PEG 4K, 15% glycerol) and flash-frozen in liquid nitrogen. Dataset was collected at the Advanced Light Source beamline 8.2.1. The dataset was indexed and integrated using iMosflm,^45^ scaled with Scala,^46^ and solved by molecular replacement using Phaser^47^ in CCP4 software suite.^48^ The crystal structure of GDP-bound K-Ras(G12C)-sotorasib adduct (PDB 6OIM) was used as the initial model. The structure was manually refined with Coot^38^ and PHENIX.^49^ Data collection and refinement statistics are listed in Table S1.

#### MOLM-13 cell experiments

The plasmids pCW57.1 (Addgene 41393), pDONR223 KRAS WT (Addgene 81751), and pDONR223 NRAS WT (Addgene 82151) were used to generate doxycycline-inducible KRAS and NRAS constructs as previously described.^23^ Briefly, mCherry was Gibson cloned from pHR-SFFV-KRAS-dCas9-P2A-mCherry (Addgene 60954) construct to the N-terminus of KRAS or NRAS on the pCW57.1 backbone. KRAS or NRAS mutagenesis on the aforementioned inducible vectors was performed with the QuikChange II XL Site-Directed Mutagenesis Kit (Agilent). Lentiviral backbone, packaging (psPAX2) and envelop (VSV-G) plasmids were transfected into 293T lenti-X cells (Takara Bio). Supernatant was collected 48 hours post-transfection and applied to MOLM-13 cells with polybrene for transduction. Cells were spin-infected at 800 g for 2 hours at 37°C. Transduced cells were treated with 2 μg/mL doxycycline (Sigma) for 24 hours and sorted for mCherry positivity. Cells were seeded into 96-well white flat bottom plates (5,000 cells/well) (Corning) and incubated for 72 h with the indicated compounds in a dilution series and 10 nM of AC220 (Selleck) and 2 μg/mL of doxycycline (Sigma) to inhibit FLT3-ITD and induce mutant Ras expression, respectively (100 μL final volume). Cell viability was assessed using a commercial CellTiter-Glo (CTG) luminescence-based assay (Promega). Briefly, the 96-well plates were equilibrated to room temperature before the addition of diluted CTG reagent (100 μL) (1:4 CTG reagent:PBS). Plates were placed on an orbital shaker for 30 min before recording luminescence using a Spark 20M (Tecan) plate reader. Independent experiments were performed three times each in technical triplicate. pERK expression levels were assessed by flow cytometry as previously described.^50,51^ Briefly, cells were seeded into 96-well round bottom plates (25,000 cells/well) (Corning) and incubated for 16 h with the indicated compounds in a dilution series and 10 nM of AC220 (Selleck) and 2 μg/mL of doxycycline (Sigma) to inhibit FLT3-ITD and induce mutant Ras expression, respectively. Fixed and permeabilized cells were stained with a pERK primary antibody (Cell Signaling Technology 4370S) for one hour, followed by a wash and incubation in a secondary antibody (Jackson ImmunoResearch 111–117-008) for one hour. Stained cells were analyzed on an Attune NxT flow cytometer (Thermo Fisher).

#### Gel electrophoresis and immunoblot

SDS-PAGE were run with Novex 4–12% or 12% Bis-Tris gel (Invitrogen) in MES running buffer (Invitrogen) at 200 V for 60 min following the manufacturer’s instructions. Protein bands were either transferred onto 0.2-μm nitrocellulose membranes (Bio-Rad) using a wet-tank transfer apparatus (Bio-Rad Criterion Blotter) in 1x TOWBIN buffer with 10% methanol at 75V for 45 min or dry transferred ultilizing the iBlot 3 0.2- μm nitrocellulose Transfer Stacks (Thermofisher) with the iBlot^™^ 3 Western Blot Transfer Device (Invitrogen). Membranes were blocked in 5% BSA–TBST for 1 h at 23°C. Primary antibody binding was performed with the indicated antibodies diluted in 5% BSA–TBST and incubated overnight at 4°C. After washing the membrane three times with TBST (5 min each wash), secondary antibodies (goat anti-rabbit IgG-IRDye 800 and goat anti-mouse IgG-IRDye 680, Li-COR) were added as solutions in 5% BSA–TBST at the dilutions recommended by the manufacturer. Secondary antibody binding was allowed to proceed for 1 h at 23°C. The membrane was washed three times with TBST (5 min each wash) and imaged on a Li-COR Odyssey fluorescence imager.

#### RalA and Rac1 activity assay

RalA and Rac1 activity was measured with either the RalA G-LISA Activation Assay (Cytoskeleton, Inc. BK129) or the Rac1 Glisa Activation Assay (Cytoskeleton, Inc. BK128), respectively, according to the vendors instruction. Briefly, HeLa cells were grown to 90% confluency in 6-well plates and transfected with 2.5 μg DNA/well using lipofectamine 3000. Cells were transfected for 24 hours then treated with DMSO or divarasib diluted in full media for 12 hours. 36 hours after transfection, cells were scraped off the 6-well plate, transferred into 1.5 ml microcentrifuge tubes, and pelleted at 800 ×g for 5 min. Medium was removed, cells were resuspended in cold PBS and pelleted again at 800 ×g for 5 min at 4°C. 100 μl of lysis buffer was added per well and the cell debris was pelleted at 6000 ×g for 4 min at 4°C. Lysate was transferred to a new tube and kept on ice. Protein concentration was measured by BCA and adjusted to 0.5 mg/ml with lysis buffer. Lysates and binding buffer were added to G-Lisa 8 well strips, incubated with antigen presenting buffer, primary, and secondary antibody, and washed in between incubation steps according to the vendor protocol. HRP detection reagent was prepared and added for 5 min. HRP stop buffer was added for 10 min before the absorbance was recorded. Absorbance was recorded using a Spark 20 M (Tecan) plate reader at λ = 492 nm.

#### Active Rap1A Detection Kit

Rap1A activity was measured with the Active Rap1A Detection Kit from Cell Signaling Technology (CST) 8818, according to the manufacturer’s instructions, and western blot analysis. Briefly, HeLa cells were grown to 90% confluency in 6-well plates and transfected with 2.5 μg DNA/well using lipofectamine 3000. Cells were transfected for 24 hours then treated with DMSO or divarasib diluted in full media for 12 hours. 36 hours after transfection, cells were scraped off the 6-well plate, transferred into 1.5 ml microcentrifuge tubes, and pelleted at 800 ×g for 5 min. Medium was removed, cells were resuspended in cold PBS and pelleted again at 800 ×g for 5 min at 4°C. Pellets were treated with 500ul of 1XLysis/Binding/Wash Buffer (Cell Signaling Technology) with 1mM PMSF. After determination of lysate protein concentration,15 μg of each lysate was prepared for western blot analysis as an input control by adding 5X Laemmli buffer and boiling at 95°C for 5 minutes. For the Rac1 pull down, 500 μg of lysate per sample was incubated with 20 μg GST-RalGDS-RBD for 1 hour at 4°C. After incubation, the samples were washed and eluted according to the manufacturer’s instructions. All samples were run on a 26 well Novex 4–12% Bis-Tris gel (Invitrogen) then prepared for western blot analysis, as described above. The primary antibodies used were the Rap1 Antibody provided with the kit and beta-Actin (CST 3700). The same secondary antibody was used as described above.

#### Rac1 downstream signaling assays

The effects of Rac1 inhibition on downstream signaling was assessed on COS7 cells via the Active Rac1 Detection Kit from Cell Signaling Technology (CST) 8818S, according to the manufacturer’s instructions, and western blot analysis. Briefly, COS7 were grown to 70% confluency in either 6-well plates or 10cm dishes and transfected for 24 hours with 2 μg DNA/well using lipofectamine 3000. Cells were then treated with divarasib or DMSO, at the indicated concentrations, in low serum media (1% FBS DMEM). After 12 hours of compound incubation, cells were placed on ice, rinsed with ice cold PBS, and either treated with 500ul of 1XLysis/Binding/Wash Buffer (Cell Signaling Technology) with 1mM PMSF for the Active Rac1 assay or 100ul of RIPA buffer (Fisher Scientific) supplemented with Halt^™^ Protease and Phosphatase Inhibitor Cocktail (Fisher Scientific), for western blot analysis. Cells were scraped and transferred to Eppendorf tubes and incubated on ice for 30 minutes with brief vortexing every 5 minutes. Lysates were then spun at 16,000 g at 4°C for 15 minutes. The supernatant was transferred to a new tube and the lysate protein concentration was determined via the Pierce^™^ Rapid Gold BCA Protein Assay (Thermo Scientific^™^).

#### Active Rac1 Detection Kit

After determination of lysate protein concentration, 15 μg of each lysate was prepared for western blot analysis as an input control by adding 5X lammeli buffer and boiling at 95°C for 5 minutes. For the Rac1 pull down, 500 μg of lysate per sample was incubated with 20 μg GST-PAK1-PBD for 1 hour at 4°C. After incubation, the samples were washed and eluted according to the manufacturer’s instructions. All samples were run on a 26 well Novex 4–12% Bis-Tris gel (Invitrogen) then prepared for western blot analysis, as described above. The primary antibodies used were the Rac1 Mouse Antibody provided with the kit, GFP (CST 2555S), and GAPDH (Proteintech 60004–1-lg). The same secondary antibody was used as described above.

#### Western blot analysis of Rac1 downstream signaling

The following primary antibodies, diluted per the manufacturers instructions, were used for for western blot analysis of downstream signaling: anti-Rac1 (Proteintech 24072–1-AP), GFP (CST 2955S), GAPDH (Proteintech 60004–1-lg), N-Cadherin (CST D4R1H), Vimentin (CST 5741), Cofilin pS3 (ab12866), Cofilin (ab2824), PAK1 (Thr423)/ PAK2 (Thr402) (CS 2601S), PAK1 (CST 2602S), P38 (Thr180/Tyr182) (CST 4511), P38 (CST 9212), MAP3K1 (Thr1400) (Proteintech 28844–1-AP), and MAP3K1 (Proteintech 19970–1-AP). The same secondary antibody was used as described above.

#### HDX-MS sample preparation

HDX reactions comparing mutant versions of Rab1A or RalA apo to Rab1A or RalA covalently bound to MRTX1257 were carried out in a 20 μl reaction volume containing 32.4 pmol of protein for RalA(G23C) and 31.2 pmol for Rab1A(S20C, E108Q). The exchange reactions were initiated by the addition of 15.9 μL of D_2_O buffer (20 mM HEPES pH 7.5, 100 mM NaCl, 94.3% D_2_O (V/V)) to 4.1 μL of protein (final D_2_O concentration of 75%). Reactions proceeded for 3s, 30s, 300s, and 3000s at 20°C before being quenched with ice cold acidic quench buffer, resulting in a final concentration of 0.6M guanidine HCl and 0.9% formic acid post quench. All conditions and timepoints were created and run in independent triplicate. Samples were flash frozen immediately after quenching and stored at −80°C until injected onto the ultra-performance liquid chromatography (UPLC) system for proteolytic cleavage, peptide separation, and injection onto a QTOF for mass analysis, described below.

#### Protein digestion and MS/MS data collection

Protein samples were rapidly thawed and injected onto an integrated fluidics system containing a HDx-3 PAL liquid handling robot and climate-controlled (2°C) chromatography system (LEAP Technologies), a Dionex Ultimate 3000 UHPLC system, as well as an Impact HD QTOF Mass spectrometer (Bruker). The full details of the automated LC system were previously described.^52^ The Rab samples were run over two immobilized pepsin columns (Waters; Enzymate Protein Pepsin Column, 300Å, 5μm, 2.1 mm × 30 mm) at 200 μL/min for 3 minutes at 2°C, while the Ral samples were run over one immobilized Nepenthesin column (Affipro; AP-PC-004 Column with immobilized Nepenthesin-2) at 200 μL/min for 3 minutes at 2°C. The resulting peptides were collected and desalted on a C18 trap column (Acquity UPLC BEH C18 1.7μm column (2.1 × 5 mm); Waters 186004629). The trap was subsequently eluted in line with an ACQUITY 1.7 μm particle, 100 × 1 mm^2^ C18 UPLC column (Waters), using a gradient of 3–35% B (Buffer A 0.1% formic acid; Buffer B 100% acetonitrile) over 11 minutes immediately followed by a gradient of 35–80% over 5 minutes. Mass spectrometry experiments acquired over a mass range from 150 to 2200 m/z using an electrospray ionization source operated at a temperature of 200°C and a spray voltage of 4.5 kV.

#### Peptide identification

Peptides were identified from the non-deuterated samples of Rab or Ral using data-dependent acquisition following tandem MS/MS experiments (0.5 s precursor scan from 150–2000 m/z; twelve 0.25 s fragment scans from 150–2000 m/z). MS/MS datasets were analyzed using FragPipe v18.0 and peptide identification was carried out by using a false discovery-based approach using a database of purified proteins and known contaminants.^53–55^ MSFragger was utilized, and the precursor mass tolerance error was set to −20 to 20ppm. The fragment mass tolerance was set at 20ppm. Protein digestion was set as nonspecific, searching between lengths of 4 and 50 aa, with a mass range of 400 to 5000 Da.

#### Mass analysis of peptide centroids and measurement of deuterium incorporation

HD-Examiner Software (Sierra Analytics) was used to automatically calculate the level of deuterium incorporation into each peptide. All peptides were manually inspected for correct charge state, correct retention time, appropriate selection of isotopic distribution, etc. Deuteration levels were calculated using the centroid of the experimental isotope clusters. Results are presented as relative levels of deuterium incorporation and the only control for back exchange was the level of deuterium present in the buffer (75%). Differences in exchange in a peptide were considered significant if they met all three of the following criteria: ≥4.5% change in exchange, ≥0.35 Da difference in exchange, and a p value <0.01 using a two tailed student t-test. Samples were only compared within a single experiment and were never compared to experiments completed at a different time with a different final D_2_O level. The data analysis statistics for all HDX-MS experiments are in Table S2 according to published guidelines.^56^ The mass spectrometry proteomics data have been deposited to the ProteomeXchange Consortium via the PRIDE partner repository^57^ with the dataset identifier PXD041440. The raw data for all analyzed peptides is available in the source data.

#### Computational modeling

Divarasib and MRTX1257 were modeled into K-Ras(G12C) based on PDB #6T5U and #6USZ separately, using Molecular Operating Environment (MOE).^58^ Key SII pocket residues involved in the K-Ras(G12C)–divarasib/MRTX1257 co-complex, R68, D69, H95, Y96 and Q99, were changed to corresponding residues in Rac1, Rab1A and Rab5C, separately, to create hybrid models with ligands bound to Rac1, Rab1A and Rab5C SII pockets in the K-Ras(G12C) structures. These hybrid models were subsequently used as templates to generate ligand bound Rac1(G12C), Rab1A(S20C) and Rab5C(S30C) homology models. The Rac1(G12C)/Rab1A(S20C)/Rab5C(S30C)– divarasib/MRTX1257 models were carefully minimized, relaxed in MD simulations, and later used to model all other ligands. For each ligand, three ligand bound non-covalent models, Rac1(G12A), Rab1A(S20A) and Rab5C(S30A), as well as three ligand bound covalent models, with ligand covalently attached to Rac1(G12C), Rab1A(S20C) and Rab5C(S30C) were generated and subjected to *in silico* testing with MD. The choice of alanine mutants in the non-covalent figure allows the evaluation of the ligand warhead positioning without clashing with cysteine Sγ. For each ligand bound model, four replicas of MD simulations were performed, and a total of 1*μ*s production simulation data was collected. Clustering analysis was conducted for each ligand bound model to obtain one or multiple representative MD protein-ligand binding poses. In addition, warhead reactivities of different ligands were evaluated using *ab initio* transition state (TS) barrier and adduct formation energy calculations. AMBER FF14SB^59^ and GAFF^60^ were applied to model the proteins and the ligands, respectively. The atomic charges of ligands were derived following the restrained electrostatic potential (RESP) procedure.^61^ Each protein-ligand system was solvated in TIP3P^62^ water, and counterions were added to neutralize the system. The particle mesh Ewald (PME) method^63^ was applied to describe the long-range electrostatics, and the SHAKE algorithm^64^ was applied to constrain hydrogen involved bonds. All MD simulations and analysis were conducted using AMBER20 (and AmberTools20) suite of programs.^65^ Visual examination of MD trajectories was performed using VMD package.^40^ QM calculations, including RESP, transition state modeling, adduct formation energies, etc., were performed using Gaussian16 software.^66^ The S-cis conformation was chosen for TS and adduct formation energy calculations, as suggested by others.^67,68^ Geometries of TS and adduct models were first optimized at HF/6–31+G** level of theory and subsequently re-optimized at B3LYP/6–31+G** level of theory, with IEF-PCM (water) method applied to model the solvation effect.

#### Bioinformatics

Multiple protein sequence alignments were conducted with Clustal Omega.^69^ Protein homology models were generated using SWISS-MODEL.^70^

#### Surface plasmon resonance

A Series S CM5 chip (Cytiva) was conditioned with a 60 second injection of 50 mM NaOH at 30 uL/min in PBS, pH 7.4. Neutravidin (Thermo Scientific) was amine-coupled to the surface in PBS using standard EDC/NHS coupling to a density of 16000–18000 RU per flow cell. Biotinylated Rap1A(2–169), RhoA(1–182) (produced at FNL) and RalA(1–206) (Amid Biosciences), were captured on individual flow cells using manual injection to a density of 2000–2400 RU in running buffer (20 mM HEPES pH 7.4, 150 mM NaCl, 2 mM MgCl2, 1 mM TCEP, 0.05% Tween-20, 5 μM GDP, 5% DMSO), with the first flow cell left empty to serve as a reference surface. A 50 mM stock of GDC-6036 in 100% DMSO was diluted 20X in DMSO-free running buffer for a final concentration of 2.5 mM in 5% DMSO. Three-fold dilutions were prepared from 833.3 μM to 1.14 μM in running buffer containing 5% DMSO. Samples were run using a multi-cycle format at 30 μL/min with 60 second contact time and 60 second dissociation time for each concentration. The experiment was conducted at 25°C on a Biacore S200 instrument (Cytiva). Sensorgrams were corrected for variations in DMSO concentration using a solvent correction cycle and double-referenced by subtracting the signal from the reference channel and a buffer blank. Sensorgram data and steady-state response values were exported into GraphPad Prism and steady-state binding curves were fit using a one-site binding model with Bmax fixed to the theoretical maximum.

#### Targeted proteomics

Proteomics experiments were performed on a TimsTOF PRO (Bruker) equipped with a CaptiveSpray source and a nanoElute system (Bruker). The peptides were separated on a 25 cm, ReproSil c18 1.5 μM 100 Å column (PepSep, PSC-25–150-15-UHP-nc). All acquired data was searched using PEAKS online Xpro 1.6 (Bioinformatics Solutions Inc). Peptide quantification was performed using Pierce quantitative peptide kit (Thermo, 23275). For SDS-PAGE gel analysis, proteins were loaded on 4–12% BisTris gels (Bolt 4–12% 17-well, Thermo, NW04127BOX). Gels were then stained with InstantBlue Coomassie protein stain (Abcam, ISB1L) and imaged using a BioRad imager (ChemiDoc XRS+ System). Protein digestion was performed using the Preomics iST kit. GFP-Trap agarose beads (ChromoTek, gta) and mini Bio-Spin columns (Bio-Rad, 7326207) were purchased. Cells overexpressing EGFP-Rac1 (G12C/K96H) were treated as indicated before they were washed with PBS three times. Cell pellets were resuspended in 1000 μL lysis buffer (10 mM Tris-HCl, 150 mM NaCl, 0.5 mM EDTA, 0.5% NP40, pH=7.5) and incubated on ice for 15 min. Cell lysates were then cleared by centrifugation at 20,000 g for 10 min at 4°C. Protein concentrations in the cleared supernatant were measured using a BCA assay kit (Pierce). Proteins were then enriched using GFP-Trap agarose beads according to the manufacturer’s instructions. In brief, 150 μL bead slurry per sample was washed with lysis buffer three times before incubation with cleared lysate for 1 h at 4°C. By using mini Bio-Spin columns, beads were washed with lysis buffer three times before 10 μL bead slurry was separated for SDS-PAGE analysis. Proteins on the washed beads were then digested using the Preomics iST kit in an on-bead digestion format according to the manufacturer’s instructions. In brief, washed beads were suspended in 100 μL LYSE buffer provided by Preomics iST kit and incubated at 55°C for 10 min for reduction and alkylation. Once the beads cooled down to room temperature, 50 μL of pre-reconstituted DIGEST were added to the beads and incubated at 37°C for 3 h with shaking. The digested peptides were then collected using mini Bio-Spin columns (Bio-Rad) and another 50 μL of LYSE buffer were added to wash the beads. Afterwards, 100 μL of STOP solution was added to the combined flow-through elution and mixed using vigorous vortexing. Then the peptides were desalted using the Preomics desalting columns before they were dried under vacuum and resuspended in 15 μL solvent A (0.1% formic acid with 2% acetonitrile) for mass spectrometry analysis. Peptide amount was monitored by quantitative colorimetric peptide assay (Pierce). Proteomics experiments were performed on a TimsTOF PRO (Bruker) equipped with a CaptiveSpray source and a nanoElute system. The peptides were separated on a 25 cm, ReproSil c18 1.5 μM 100 Å column (PepSep, PSC-25–150-15-UHP-nc) using a step-wise linear gradient method with water in 0.1% formic acid (solvent A) and acetonitrile with 0.1% formic acid (solvent B): 5–30% solvent B for 90 min at 0.5 μL/min, 30–35% solvent B for 10 min at 0.6 μL/min, 35–95% solvent B for 4 min at 0.5 μL/min, 95% hold for 4 min at 0.5 μL/min). Acquired data was collected in a data-dependent acquisition mode with ion mobility activated in PASEF mode. MS and MS/MS spectra were collected with m/z ranging from 100 to 1700 in positive mode. All acquired data was searched using PEAKS online Xpro 1.6 (Bioinformatics Solutions Inc.). Spectral searches were performed using a custom FASTA-formatted dataset containing EGFF-Rac1 (G12C/K96H) and common contaminant. A precursor mass error tolerance was set to 20 ppm and a fragment mass error tolerance was set at 0.03 ppm. Peptides, ranging from 6 to 45 amino acids in length, were searched in specific trypsin digest mode with a maximum of one missed cleavage. Carbamidomethylation (+57.0214 Da) on cysteines was set as a static modification while methionine oxidation (+15.9949 Da), lysine acetylation (+42.0115 Da) and divarasib (+621.2242 Da) were set as a variable modification. Peptides were filtered based on a false discovery rate (FDR) of 1%. Proteomics data were analyzed by PEAKS online (Xpro 1.6). Images were made using Adobe Illustrator (v22.1).

### QUANTIFICATION AND STATISTICAL ANALYSIS

All of the curves in figures were fitted by GraphPadPrism. All of the details can be found in the figure legends and in the method details. The data collection and refinement statistics of the crystal structures can be found in Table S1.

## Supplementary Material

MMC1

MMC2

1

## Figures and Tables

**KEY RESOURCES TABLE:** 

REAGENT or RESOURCE	SOURCE	IDENTIFIER

Antibodies		
M-Ras	Abcam	Ab176570
R-Ras	Abcam	Ab191399
Ras	Abcam	Ab108602
Rheb	CST	1379S
RalA	Abcam	Ab126627
Rap1A	Abcam	Ab96223
N-cadherin	CST	D4R1H
Vimentin	CST	5741S
Cofilin (pS3)	Abcam	Ab12866
Cofilin	Abcam	Ab42824
PAK1 (pThr423)/Pak2 pThr402	CST	2601S
PAK1	CST	2692S
Rac1	Proteintech	24072–1-AP
GFP	CST	2955S
P38 (pThr180/pTyr182)	CST	4511S
P38	CST	9212S
MAP3K1 (pThr1400)	Proteintech	28844–1-AP
MAP3K1	Proteintech	19970–1-AP
GAPDH	Proteintech	60004–1-lg
beta-Actin	CST	4967S
IRDye 800CW Goat anti-Human	LI-COR	926–32211
IRDye 680RD Goat anti-Human	LI-COR	926–68070
Bacterial and virus strains		
*Escherichia coli* BL21(DE3)	NEB	Cat# C2527H
Chemicals, peptides, and recombinant proteins		
GDP	Sigma-Aldrich	Cat# G7127–100MG
Guanosine 5’-[β,γ-imido]triphosphate (GNP, GppNHp)	Axorra	Cat# JBS-NU-401–50
GTPγS	Abcam	Cat# ab146662
RAPGEF5	Cytoskeleton	Cat# CS-GE09
His-Rac1(WT)	Cytoskeleton	Cat# RC01
His-RabL5(WT)	Abcam	Cat# ab156964
His-Rab1A(WT)	Abcam	Cat# ab90975
Biotinylated Avi-Rap1A(WT)	This study	N/A
Biotinylated Avi-RhoA(WT)	This study	N/A
Biotinylated Avi-RalA(WT)	Amid Biosciences	Cat# RALA-B-301
Phospho p44/42 MAPK ERK1/2 (Thr202 Tyr204) (D13.14.4E)	Cell Signaling Technology	Cat# 4370S
R-PE-Fab Goat Anti-Rabbit IgG, Fc Secondary Antibody	Jackson ImmunoResearch	Cat# 111–117-008
EDTA 0.5 M in water, pH 8.0	Sigma-Aldrich	Cat# 03690
Sypro Orange	Thermo Fisher Scientific	S6650
TEV Protease	Sigma-Aldrich	Cat# T4455
cOmplete Protease Inhibitor Cocktail	Sigma-Aldrich	Cat# 5056489001
Carbenicillin	Goldbio	Cat# C-103–100
Kanamycin	Goldbio	Cat# K-120–25
IPTG	Goldbio	Cat# I2481C100
DTT	Goldbio	Cat# DTT10
1M HEPES pH 7.4	Teknova	Cat# H1030
1M Magnesium Chloride	Thermo Fisher Scientific	Cat# AM9530G
5M Sodium Chloride	Sigma-Aldrich	Cat# S6546–4L
10% Tween-20	Bio-Rad	Cat# 1610781
Bond-Breaker TCEP Solution, Neutral pH	Thermo Fisher Scientific	Cat# 77720
NeutrAvidin Protein	Thermo Fisher Scientific	Cat# 31000
ProteOn Sodium Hydroxide Solution, 50mM	Bio-Rad	Cat# 1762230
10X RIPA buffer	EMD Millipore	20–188
4X LDS sample loading buffer	G Biosciences	786–323
cOmplete protease inhibitor cocktail	Roche	45–11836170001
InstantBlue Coomassie Protein Stain	Abcam	Ab119211
GFP-Trap agarose beads	ChromoTek	gta
Sotorasib (AMG510)	Cayman	Cat# 29465
Adagrasib (MRTX849)	Cayman	Cat# 31440
MRTX1257	Cayman	Cat# 33527
ARS853	Cayman	Cat# 19137
ARS1620	Cayman	Cat# 27915
GDC6036	MedChemExpress	Cat# HY-145928
JDQ443	MedChemExpress	Cat# HY-139612
Garsorasib	MedChemExpress	Cat# HY-145571
ASP2453	MedChemExpress	Cat# HY-132966
RMC-6291	MedChemExpress	Cat# HY-153346
Compound 11	Medicilon Inc	Custom Order
Compound 12	Medicilon Inc	Custom Order
Compound 13	Medicilon Inc	Custom Order
Compound 14	Medicilon Inc	Custom Order
Compound 15	Medicilon Inc	Custom Order
Compound 16	Medicilon Inc	Custom Order
Compound 17	Medicilon Inc	Custom Order
Compound 18	Medicilon Inc	Custom Order
Compound 19	Medicilon Inc	Custom Order
Compound 20	Medicilon Inc	Custom Order
Compound 21	Medicilon Inc	Custom Order
Compound 22	Medicilon Inc	Custom Order
Deuterium Oxide 99.9%	Sigma Aldrich	151882–10X1ML
Critical commercial assays		
RalA G-LISA Activation Assay Kit 96 Assays	Cytoskeleton	Cat# BK129
Rac1 G-LISA Activation Assay Kit 96 Assays	Cytoskeleton	Cat# BK128
Active Rap1 Detection Kit	Cell Signaling Technology	Cat# 8818
Active Rac1 Detection Kit	Cell Signaling Technology	Cat#8815S
Quantitative colorimetric peptide assay	Pierce	23275
Quantitative protein BCA assay	Pierce	23225
PreOmics iST kit	PreOmics	P.O.00027
CellTiter-Glo	Promega	G7570
Deposited data		
AMG510-bound H-Ras(G12C)	This study	PDB 8TLR
Mass spectrometry proteomics data	PRIDE	PXD041440 and PXD054414
Experimental models: Cell lines		
HeLa	ATCC	Cat# CCL-2
MOLM-13	DSMZ	Cat# ACC 554
MOLM-13 Inducible NRAS G12C	This study	N/A
MOLM-13 Inducible KRAS G12C	This study	N/A
MOLM-13 Inducible KRAS G12D	This study	N/A
COS-7	Gifted by the Jura lab	N/A
Recombinant DNA		
pProEx H-Ras(G12C) CL	This study	Addgene #224261
pProEx N-Ras(G12C) CL	This study	Addgene #224262
pProEx RalA(G23C) CL	This study	Addgene #224263
pProEx Rap1A(G12C) CL	This study	N/A
pProEx Rap1A(G12C, L96F) CL	This study	Addgene #224264
pProEx Ypt1(S17C) CL	This study	Addgene #224265
pProEx Rheb(R15C) CL	This study	Addgene #224266
pProEx Rit1(G47C) CL	This study	Addgene #224267
pProEx M-Ras(G22C) CL	This study	Addgene #224268
pcDNA3.1(+) EGFP-RalA(WT)	This study	Addgene #224274
pcDNA3.1(+) EGFP-RalA(G23C)	This study	Addgene #224275
pcDNA3.1(+) EGFP-Rap1A(WT)	This study	Addgene #224276
pcDNA3.1(+) EGFP-Rap1A(G12C, L96F)	This study	Addgene #224277
pProEx Rac1(G12C) CL	This study	Addgene #224269
pProEx Rac1(G12C, W97Y) CL	This study	N/A
pProEx Rac1(G12C, E100Q) CL	This study	N/A
pProEx Rac1(G12C, K96H) CL	This study	Addgene #224270
pProEx Rac1(G12C, P29S) CL	This study	N/A
pProEx RhoA(G14C) CL	This study	Addgene #224271
pProEx Rab1A(S20C) CL	This study	Addgene #224272
pProEx Rab1A(S20C, E108Q) CL	This study	N/A
pProEx Rab5C (S30C) CL	This study	Addgene #224273
pcDNA3.1(+) EGFP-Rac1 (WT)	This study	Addgene #224278
pcDNA3.1(+) EGFP-Rac1 (G12C)	This study	Addgene #224279
pcDNA3.1(+) EGFP-Rac1 (G12C, K96H)	This study	Addgene #224281
pcDNA3.1(+) EGFP-Rac1 (P29S)	This study	Addgene #224280
pcDNA3.1(+) EGFP-Rac1 (G12C, P29S, K96H)	This study	Addgene #224282
pcDNA3.1(+) K-Ras(WT)	This study	Addgene #224283
pcDNA3.1(+) K-Ras(G12C)	This study	Addgene #224284
pcDNA3.1(+) R-Ras1(WT)	This study	Addgene #224285
pcDNA3.1(+) R-Ras1(G38C)	This study	Addgene #224286
pcDNA3.1(+) M-Ras(WT)	This study	Addgene #224287
pcDNA3.1(+) M-Ras(G22C)	This study	Addgene #224288
pcDNA3.1(+) Rheb(WT)	This study	Addgene #224289
pcDNA3.1(+) Rheb(R15C)	This study	Addgene #224290
NRAS(G12C) in pCW57.1 vector	This study	N/A
KRAS(G12C) in pCW57.1 vector	This study	N/A
KRAS(G12D) in pCW57.1 vector	This study	N/A
Avi-RhoA(WT) in pDest-566 vector	This study	N/A
Avi-Rap1A(WT) in pDest-566 vector	This study	N/A
Software and algorithms		
GraphPad Prism	GraphPad Software	https://www.graphpad.com/scientific-software/prism/
Phenix	Adams et al.^37^	https://www.phenix-online.org/
Coot	Emsley et al.^38^	https://www2.mrc-lmb.cam.ac.uk/personal/pemsley/coot/
Excel	Microsoft	https://www.microsoft.com/en-us/
ChemDraw	Revvity Signals	https://www.revvitysignals.com
SnapGene	Dotmatics	https://www.snapgene.com/
Zotero 5.0	Corporation for Digital Scholarship	https://www.zotero.org/
Word	Microsoft	https://www.microsoft.com/en-us/
Illustrator 2022	Adobe	https://www.adobe.com/products/illustrator.html
AMBER20	Case et al.^39^	https://ambermd.org/
Gaussian16	Gaussian	https://gaussian.com/gaussian16/
VMD	Humphrey et al.^40^	https://www.ks.uiuc.edu/Research/vmd/
Pymol	The PyMOL Molecular Graphics System; Version 1.8; Schrodinger, LLC	https://pymol.org/2/
HDExaminer	Sierra Analytics	http://massspec.com/hdexaminer
Bruker Compass DataAnalysis 4.2	Bruker	https://www.bruker.com
FragPipe (v19.1)	Nesvizhskii Lab - University of Michigan	https://fragpipe.nesvilab.org/
ImageStudioLite	Li-Cor	Version 5.2.5
PEAKS online	Bioinformatics Solutions Inc.	Xpro 1.6
Biacore S200 Control Software	Cytiva	Version 1.1.1
Biacore S200 Evaluation Software	Cytiva	Version 1.1.1
Other		
TALON Metal Affinity Resin	Clontech Laboratories	Cat# 635503
Superdex 75 Increase 10/300 GL	Cytiva	Cat# 29148721
Zeba Spin Desalting Columns, 7K MWCO	Thermo Fisher Scientific	Cat# 89882
Slide-A-Lyzer Dialysis Cassettes, 10K MWCO	Thermo Fisher Scientific	Cat# 66380
Pierce Protein Concentrators, 10 K MWCO, 15 mL	Thermo Fisher Scientific	Cat# 88528
Pierce Protein Concentrators, 10 K MWCO, 5 mL	Thermo Fisher Scientific	Cat# 88517
Lipofectamine 3000 Transfection Reagent	Thermo Fisher Scientific	Cat# L3000015
NuPAGE 4 to 12%, Bis-Tris, Protein Gels	Thermo Fisher Scientific	Cat# NP0322BOX
Spark 20 M plate reader	TECAN	Cat# 20173336– 03
C18 trap column	Waters	186003975
Pepsin column	Waters	186007233
Nepenthesin Column	Affipro	AP-PC-004
C18 UPLC column	Waters	186005593
Mini Bio-Spin column	Bio-Rad	7326207
10X RIPA buffer	EMD Millipore	20–188
4X LDS sample loading buffer	G Biosciences	786–323
InstantBlue Coomassie Protein Stain	Abcam	Ab119211
GFP-Trap agarose beads	ChromoTek	gta
Amine Coupling Kit	Cytiva	Cat# BR100050
Series S CM5 Chips	Cytiva	Cat# 29149603
iBlot 3 Transfer Stacks, midi nitrocellulose	Thermo Scientific	IB33001X3
iBlot 3 Western Blot Transfer Device	Thermo Scientific	IB31001

## Highlights

- Druggable switch II pocket is present in many GTPases beyond K-Ras
- Ligand-bound H-Ras, RalA, and Rab1A were characterized by X-ray or HDX-MS
- Inhibition of GTPase function is demonstrated *in vitro* and in cells
- Switch II pocket inhibitor analogs show improved labeling of Rho and Rab GTPases
