# Supplementary material for: Targeting Ras-, Rho-, and Rab-family GTPases via a conserved cryptic pocket
Source: Cell. Author manuscript; Available in PMC 2024 Nov 3. (PMC11531380; doi:10.1016/j.cell.2024.08.017)
Supplement: MMC1 [file NIHMS2023477-supplement-MMC1.pdf]

Cell, Volume *187*

Supplemental information

Targeting Ras-, Rho-, and Rab-family GTPases via a conserved cryptic pocket

Johannes Morstein, Victoria Bowcut, Micah Fernando, Yue Yang, Lawrence Zhu, Meredith L. Jenkins, John T. Evans, Keelan Z. Guiley, D. Matthew Peacock, Sophie Krahnke, Zhi Lin, Katrine A. Taran, Benjamin J. Huang, Andrew G. Stephen, John E. Burke, Felice C. Lightstone, and Kevan M. Shokat

Table S1. X-ray crystallography data collection and refinement statistics. Related to Figure 1.

Values in parentheses are for highest resolution shell.

	**H-Ras (G12C)-sotorasib**
Data collection	
Space group	$H3_2$
Cell dimensions	
a, *b*, *c* (Å)	92.35, 92.35, 119.58
α, β, γ	90, 90, 120
Resolution (Å)	66.48-1.7 (1.73 – 1.7)
R_{merge}	0.052 (0.133)
R_{pim}	0.036 (0.096)
$I / \sigma I$	15.8 (6.8)
CC 1/2	0.978 (0.560)
Total reflections	102754 (5419)
Unique Reflections	21497 (1122)
Completeness (%)	99.1 (100.0)
Redundancy	4.8 (4.8)
Refinement	
Resolution (Å)	33.28-1.7
No. reflections	21560 (2158)
R_{work} / R_{free}	18.41/20.91
No. atoms	1439
Protein	1290
Water	107
Refined B-factors (Å^2)	
Overall	18.66
Compound	21.83
water	25.67
R.m.s. deviations	
Bond lengths (Å)	0.008
Bond angles (°)	1.00
Ramachandran analysis	
Favored (%)	98.7
Disallowed (%)	0

Table S2. HDX-MS Statistics. Related to Figure 3 and Figure 5.

Data set	**RalA Apo**	**RalA – MRTX1257**	**Rab1a Apo**	**Rab1a - MRTX1257**
HDX reaction details	%D_2O=75%<br>$pH_{(read)}$=7.5<br>Temp=20ºC	%D_2O=75%<br>$pH_{(read)}$=7.5<br>Temp=20ºC	%D_2O=75%<br>$pH_{(read)}$=7.5<br>Temp=20ºC	%D_2O=75%<br>$pH_{(read)}$=7.5<br>Temp=20ºC
HDX time course (seconds)	3s,30s,300s,3000s	3s,30s,300s,3000s	3s,30s,300s,3000s 3000s	3s,30s,300s,3000s
HDX controls	N/A	N/A	N/A	N/A
Back-exchange	No correction, deuterium levels are relative	No correction, deuterium levels are relative	No correction, deuterium levels are relative	No correction, deuterium levels are relative
Number of peptides	51	51	54	54
Sequence coverage	87.9%	87.9%	88.8%	88.8%
Average peptide /redundancy	Length= 9.1<br>Redundancy= 2.6	Length= 9.1<br>Redundancy= 2.6	Length= 12.3<br>Redundancy= 2.8	Length= 12.3<br>Redundancy= 2.8
Replicates	3	3	3	3
Repeatability	Average StDev=0.9%	Average StDev=0.9%	Average StDev=0.9%	Average StDev=0.7%
Significant differences in HDX	>4.5% and >0.35 Da and unpaired t-test ≤0.01	>4.5% and >0.35 Da and unpaired t-test ≤0.01	>4.5% and >0.35 Da and unpaired t-test ≤0.01	>4.5% and >0.35 Da and unpaired t-test ≤0.01
