# Supplementary material for: Targeting Ras-, Rho-, and Rab-family GTPases via a conserved cryptic pocket
Source: Cell. Author manuscript; Available in PMC 2024 Nov 3. (PMC11531380; doi:10.1016/j.cell.2024.08.017)
Supplement: 1 [file NIHMS2023477-supplement-1.pdf]

CellPress
OPEN ACCESS

Supplemental figures

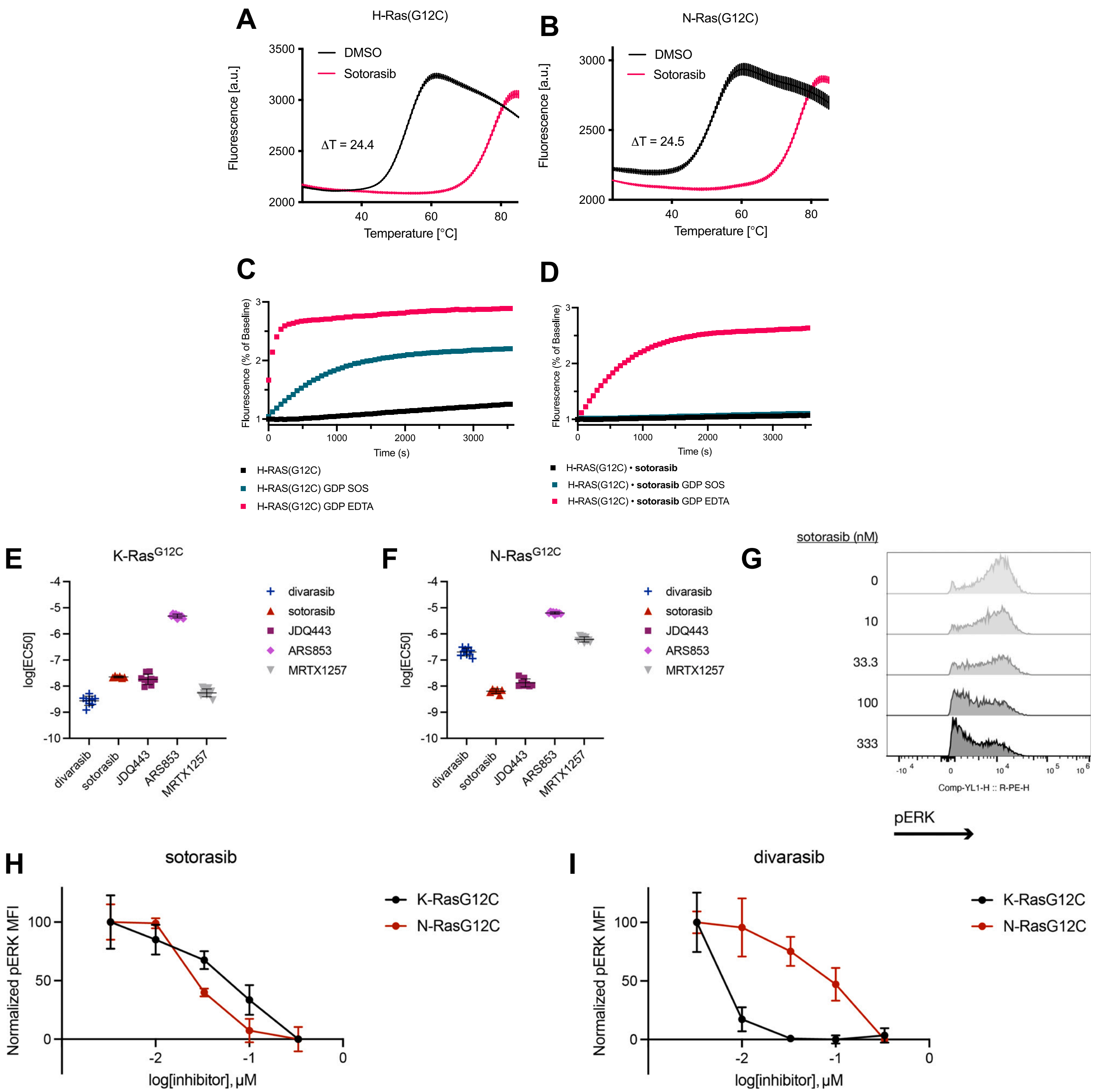

Figure S1. H-Ras(G12C) *in vitro* characterization and cellular inhibition of N-Ras(G12C), related to Figure 1
(A) Differential scanning fluorimetry of H-Ras(G12C)•GDP and H-Ras(G12C)•GDP•sotorasib adduct.
(B) Differential scanning fluorimetry of N-Ras(G12C)•GDP and N-Ras(G12C)•GDP•sotorasib adduct.
(C) Intrinsic, SOS-, or EDTA-mediated nucleotide exchange of BODIPY-GDP with N-Ras(G12C)•GDP.
(D) Intrinsic, SOS-, or EDTA-mediated nucleotide exchange of BODIPY-GDP with N-Ras(G12C)•GDP•sotorasib adduct.
(E and F) Quantification of relative growth of MOLM-13-KRAS-G12C (E) and MOLM-13-NRAS-G12C (F) cells after treatment with K-Ras(G12C) inhibitors for 72 h. Data are presented as mean ±SD (n = 3) and are representative of three independent experiments.

(legend continued on next page)

(G) Phospho-flow experiment flow cytometry data of MOLM-13-NRAS-G12C treated with increasing concentrations of sotorasib.
(H) Dose-dependent pERK inhibition in MOLM-13-KRAS-G12C and MOLM-13-NRAS-G12C cells treated with increasing concentrations of sotorasib measured by phospho-flow assay.
(I) Dose-dependent pERK inhibition in MOLM-13-KRAS-G12C and MOLM-13-NRAS-G12C cells treated with increasing concentrations of divarasib measured by phospho-flow assay. Data are presented as mean ± SD (n = 3) and are representative of three independent experiments.

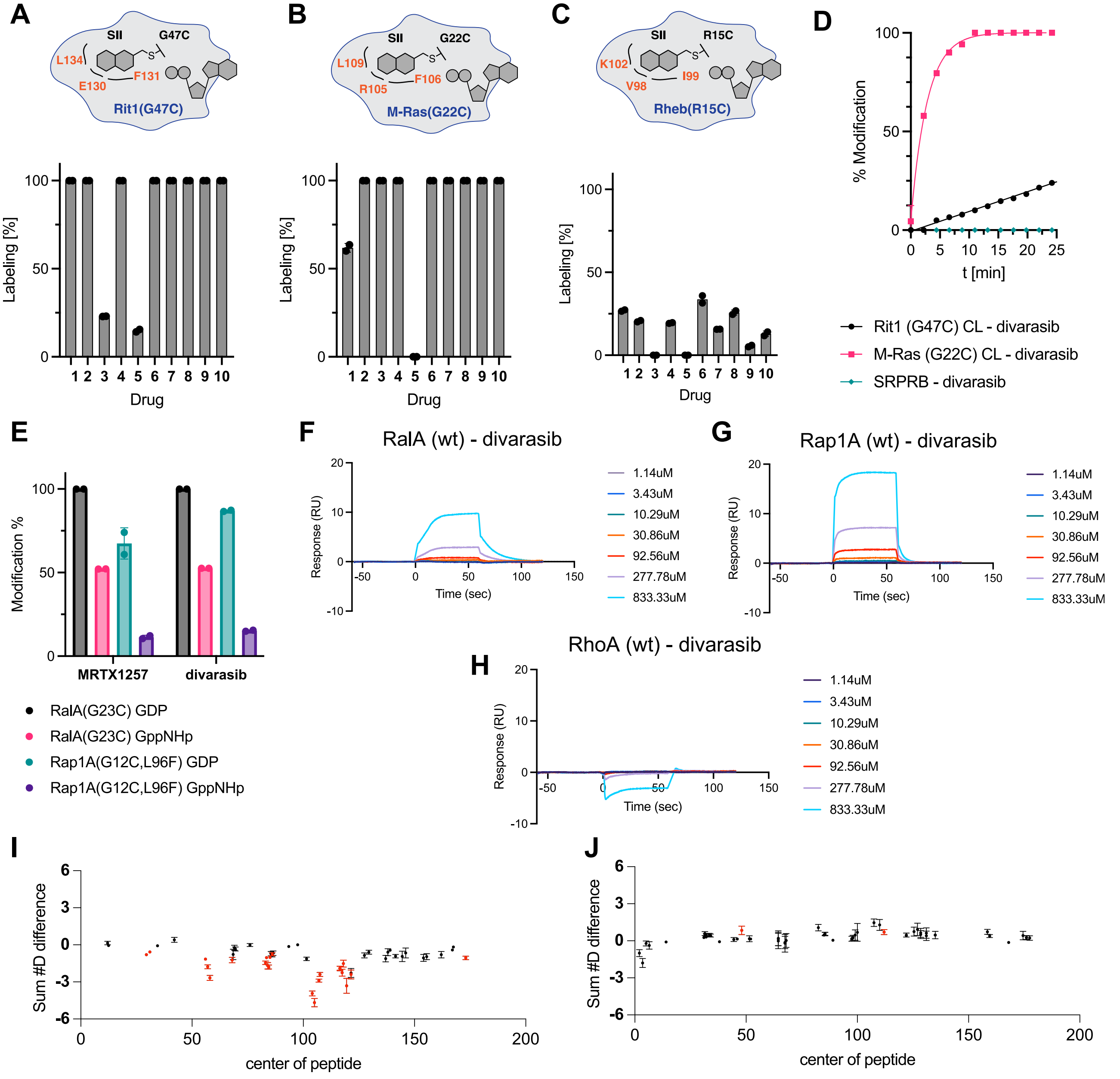

Figure S2. Covalent engagement of additional Ras-family GTPases and GTP-bound Ras-family GTPases and reversible binding, related to Figures 2, 3, and 5

(A) Covalent modification of Rit1(G47C) with compounds **1–10** (50 µM, 12 h).

(B) Covalent modification of M-Ras(G22C) with compounds **1–10** (50 µM, 12 h).

(C) Covalent modification of Rheb(R15C) with compounds **1–10** (50 µM, 12 h).

(D) Time-dependent covalent modification of Rit1(G47C), M-Ras(G22C), and SRPRB with divarasib (50 µM).

(E) Covalent modification of RalA(G23C)•GDP, Rap1A(G12C, L96F)•GDP, RalA(G23C)•GppNHp, and Rap1A(G12C, L96F)•GppNHp with MRTX1257 and divarasib (50 µM, 1 h).

(F–H) Surface plasmon resonance experiment with divarasib and RalA(WT), Rap1A(WT), and RhoA(WT).

(I and J) All HDX-MS peptide changes #D for experiments examining changes in dynamics caused by binding of MRTX1257. (I) The sum of the number of deuteron difference for all peptides analyzed over the entire deuterium exchange time course for RalA(G23C)•GDP and RalA(G23C)•GDP•MRTX1257. (J) The sum of the number of deuteron difference for all peptides analyzed over the entire deuterium exchange time course for Rab1A(S20C,E108Q)•GDP and Rab1A(S20C,E108Q)•GDP•MRTX1257. Peptides colored in red are those that had a significant change (>0.35 Da and 4.5% difference at any time point, with a two-tailed t test $p < 0.01$). Each point represents a single peptide, and error bars are shown as the sum of SD across all time points ($n = 3$ for each time point).

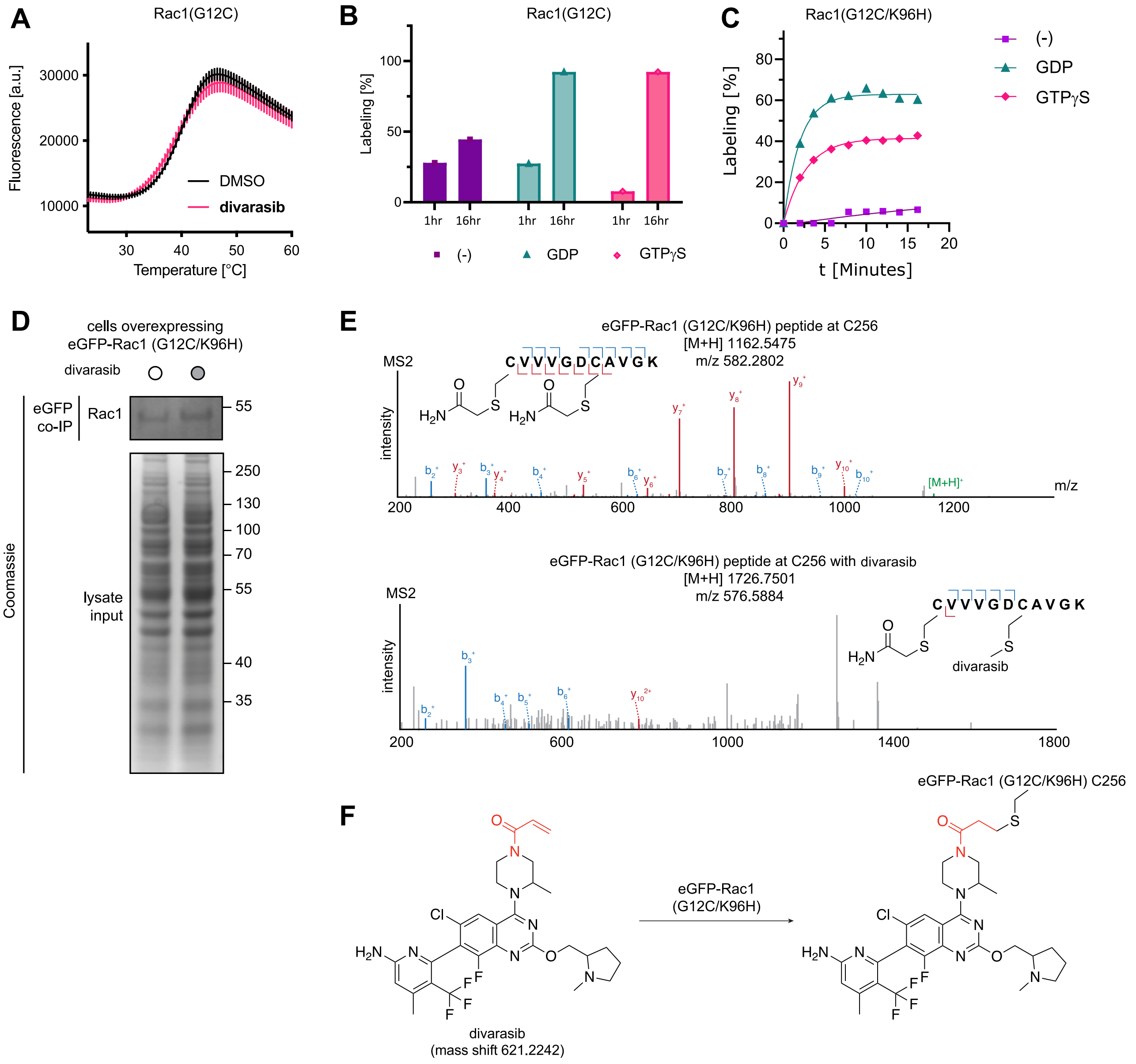

Figure S3. Further characterization of Rac1 targeting, related to Figure 5

(A) Differential scanning fluorimetry of Rac1(G12C)•GDP and Rac1(G12C)•GDP•divarasib adduct.

(B) Covalent modification of Rac1(G12C), Rac1(G12C)•GDP, and Rac1(G12C)•GTPγS with divarasib (50 μM, 2 h).

(C) Time-dependent covalent modification of Rac1(G12C,K96H), Rac1(G12C,K96H)•GDP, and Rac1(G12C,K96H)•GTPγS with divarasib (50 μM).

(D) SDS-PAGE analysis of EGFP-Rac1(G12C,K96H) enrichment from HeLa lysate. Prior to lysis, cells were transiently transfected with EGFP-Rac1(G12C,K96H) and treated with or without divarasib (20 μM) for 6 h. Protein was enriched using GFP-Trap beads.

(E) Divarasib covalently modifies C256 in EGFP-Rac1(G12C,K96H), which corresponds to G12 of Rac1(G12C,K96H). Site of modification was verified by LC-MS/MS following digest of the enriched lysates. Representative spectra of peptides from treated and untreated sample are shown.

(F) Structure of presumed adduct of divarasib with C256 of EGFP-Rac1(G12C,K96H).

CellPress
OPEN ACCESS

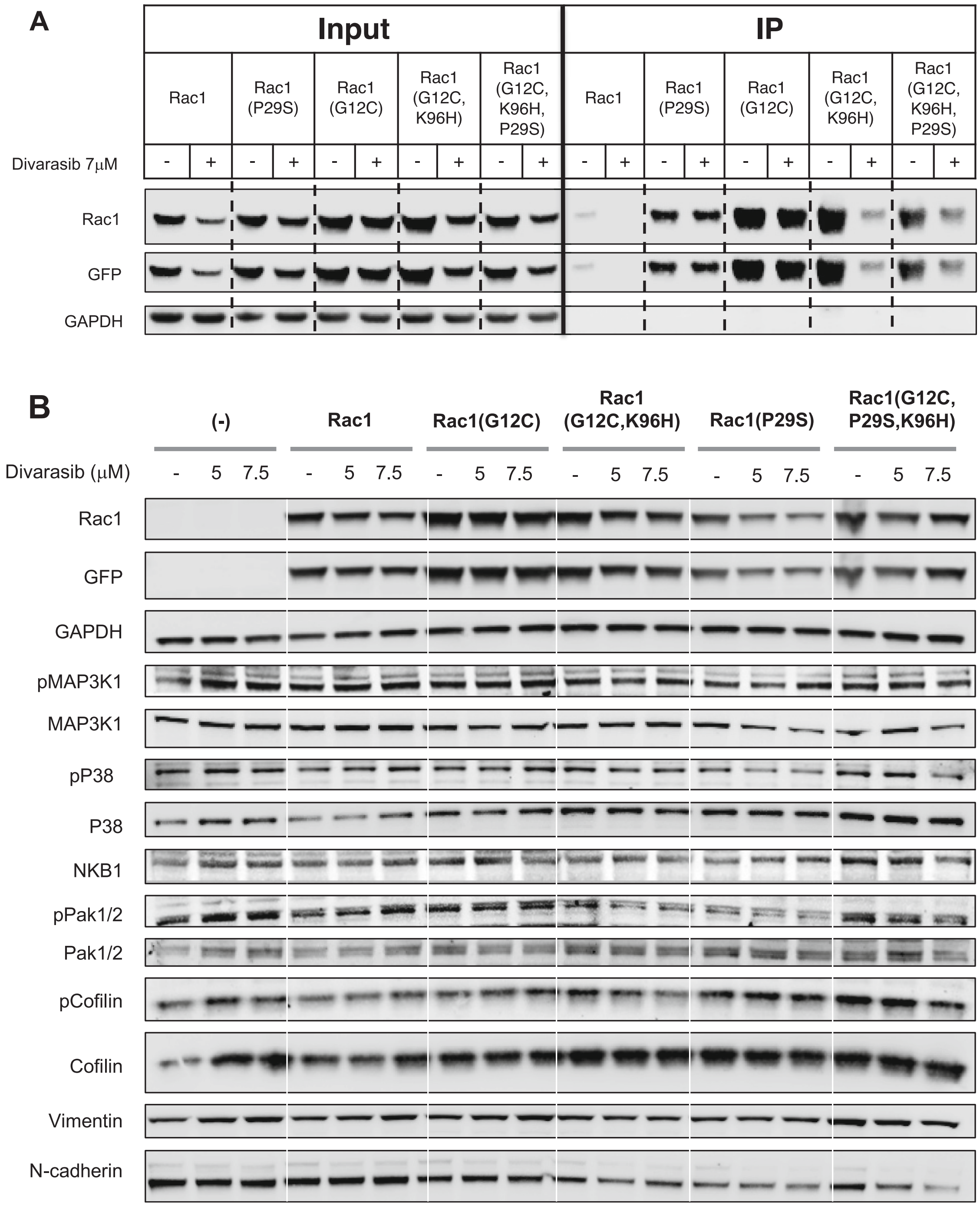

Figure S4. Cellular inhibition of Rac1 mutants with divarasib, related to Figure 5

(A) IP of active GTP-bound Rac1 using GST-PAK1-RBD of COS7 cells transiently overexpressing EGFP-Rac1(WT), Rac1(P29S), Rac1(G12C), Rac1(G12C,K96H), and Rac1(G12C,K96H,P29S) and treated with divarasib.

(B) Western blot of COS7 cells transiently overexpressing EGFP-Rac1(WT), Rac1(P29S), Rac1(G12C), Rac1(G12C,K96H), and Rac1(G12C,K96H, P29S) in low-serum conditions and treated with divarasib.

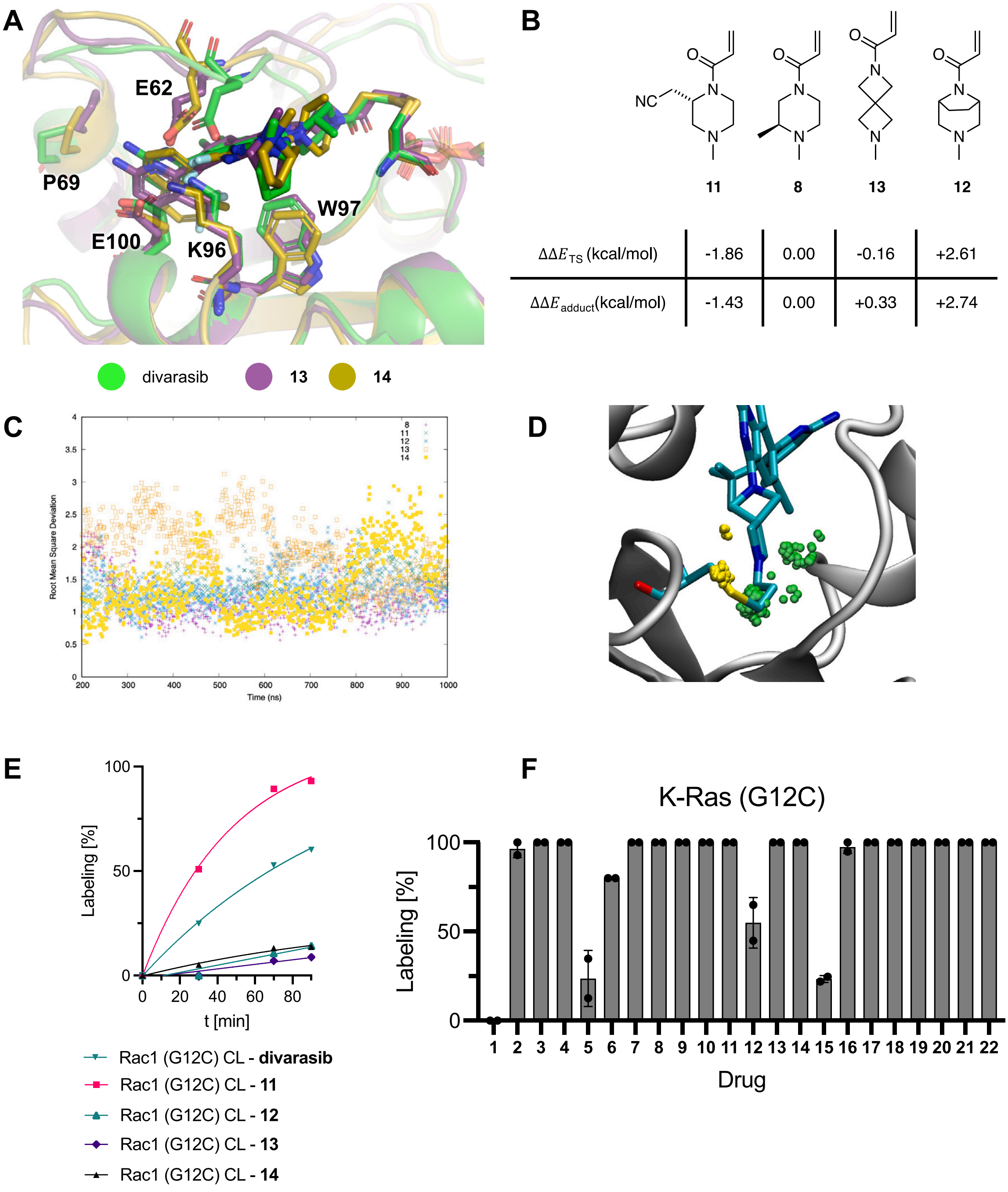

Figure S5. Computational modeling and covalent engagement of Rac1(G12C) and K-Ras(G12C) with extended compound library, related to Figure 6

(A) **8**, **13**, and **14** sample different binding conformation in covalent Rac1(G12C) MD simulations. (B) QM (B3LYP/6-31+G** IEF-PCM) calculations of relative TS barriers and covalent adduct energies with the respective warhead motifs (to **8**). **11**, **13**, and **14** are predicted to have higher, comparable, and lower intrinsic reactivities compared with **8**, respectively.

(C) **13** exhibits significant larger root mean square deviation on ligand-heavy atoms than **8**, **11**, and **12** in non-covalent Rac1(G12C) MD simulations, suggesting compromised stability of the Rac1(G12C)-**13** complex.

(D) Unideal warhead placement thus potential impaired covalent modification is suggested by comparing of G12C Sγ (yellow beads) positioning from Rac1(G12C)-**13** covalent MD simulations with the warhead-reacting C atom (green beads) positioning from Rac1(G12C)-**13** non-covalent MD simulations, projecting onto the initial covalent model of **13** (sticks) bound Rac1(G12C) (gray cartoon).

(E) Time-dependent covalent modification of Rac1(G12C) with divarasib and 11–14 (50 μM).

(F) Covalent modification of K-Ras(G12C) with compounds **1–22** (50 μM, 30 s).

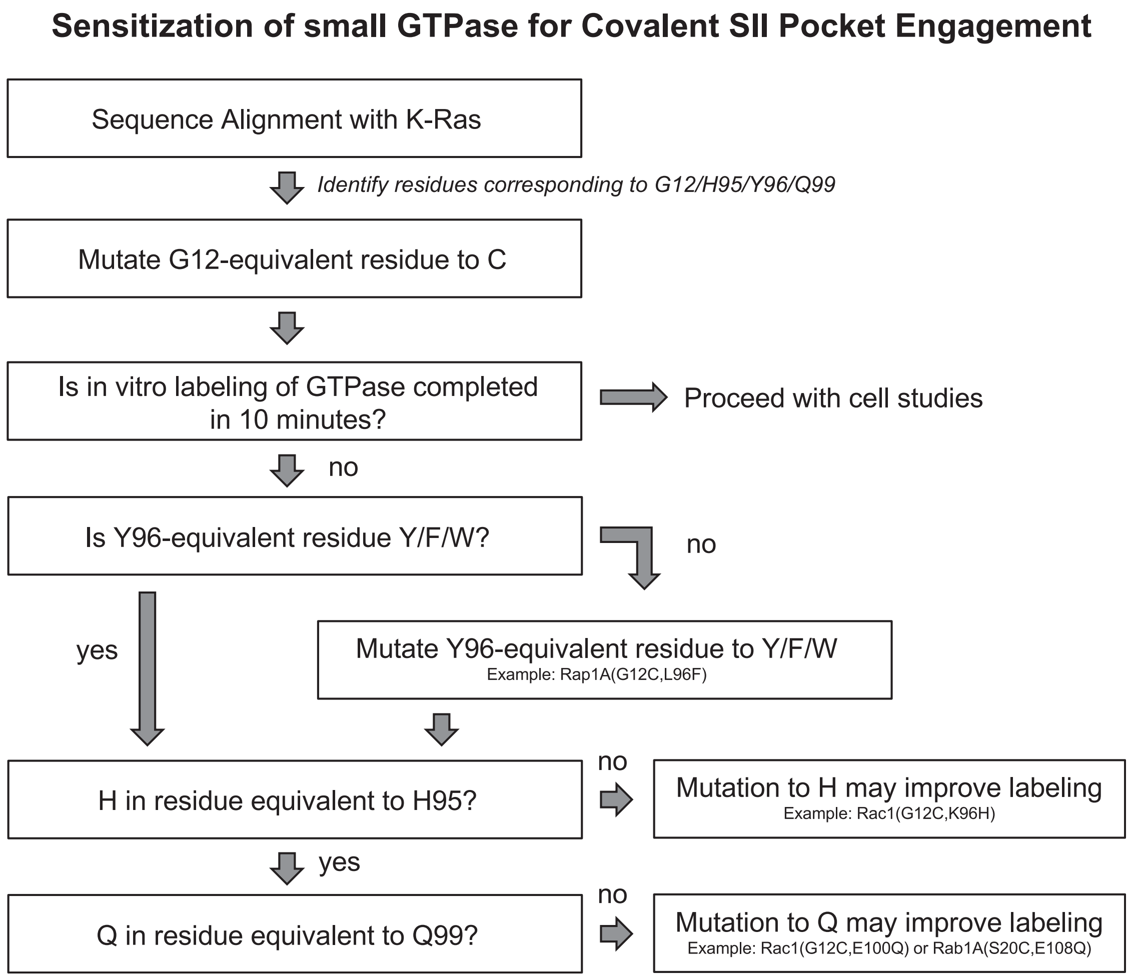

Figure S6. Scheme for sensitization of small GTPases for covalent SII pocket engagement, related to Figure 6
